# Are side effects of cannabidiol (CBD) products caused by tetrahydrocannabinol (THC) contamination?

**DOI:** 10.12688/f1000research.19931.3

**Published:** 2020-08-19

**Authors:** Dirk W. Lachenmeier, Stephanie Habel, Berit Fischer, Frauke Herbi, Yvonne Zerbe, Verena Bock, Tabata Rajcic de Rezende, Stephan G. Walch, Constanze Sproll

**Affiliations:** 1 Chemisches und Veterinäruntersuchungsamt (CVUA) Karlsruhe, Karlsruhe, 76187, Germany

**Keywords:** Tetrahydrocannabinol, cannabidiol, Cannabis sativa, hemp, food supplements, risk assessment, drug effects

## Abstract

Cannabidiol (CBD)-containing products are widely marketed as over the counter products, mostly as food supplements, to avoid the strict rules of medicinal products. Side-effects reported in anecdotal consumer reports or during clinical studies were first assumed to be due to hydrolytic conversion of CBD to psychotropic Δ ^9^-tetrahydrocannabinol (Δ ^9^-THC) in the stomach after oral consumption. However, research of pure CBD solutions stored in simulated gastric juice or subjected to various storage conditions such as heat and light with specific liquid chromatographic/tandem mass spectrometric (LC/MS/MS) and ultra-high pressure liquid chromatographic/quadrupole time-of-flight mass spectrometric (UPLC-QTOF) analyses was unable to confirm THC formation. Another hypothesis for the side-effects of CBD products may be residual Δ ^9^-THC concentrations in the products as contamination, because most of them are based on crude hemp extracts containing the full spectrum of cannabinoids besides CBD. Analyses of 67 food products of the German market (mostly CBD oils) confirmed this hypothesis: 17 products (25%) contained Δ ^9^-THC above the lowest observed adverse effects level (2.5 mg/day). Inversely, CBD was present in the products below the no observed adverse effect level. Hence, it may be assumed that the adverse effects of some commercial CBD products are based on a low-dose effect of Δ ^9^-THC and not due to effects of CBD itself. The safety, efficacy and purity of commercial CBD products is highly questionable, and all of the products in our sample collection showed various non-conformities to European food law such as unsafe Δ ^9^-THC levels, full-spectrum hemp extracts as non-approved novel food ingredients, non-approved health claims, and deficits in mandatory food labelling requirements. In view of the growing market for such lifestyle products, the effectiveness of the instrument of food business operators' own responsibility for product safety must obviously be challenged.

## Introduction

Since hemp has been re-approved for cultivation as an industrial crop in the form of low Δ ^9^-tetrahydrocannabinol (∆ ^9^-THC) hemp varieties in the European Union, components of the hemp plant are increasingly used for the production of foods and other consumer products such as liquids for electronic cigarettes ^1^.

From all hemp constituents, cannabidiol (CBD) is currently the compound with highest interest. In contrast to ∆ ^9^-THC, the major drug-constituent of hemp, CBD is a non-psychotropic cannabinoid. It is currently being tested for its possible antispasmodic, anti-inflammatory, anxiolytic and antiemetic effects as a drug, e.g. for the treatment of epilepsy ^2, 3^. However, CBD products of all kinds can now also be purchased in organic shops, drug stores, supermarkets and via the Internet, mostly by advertising dubious “cure-all” properties including anti-carcinogenic effects or various unspecific health advantages. The marketing of CBD products is based on the current “hype” around medicinal hemp products, whereby the CBD products are offered as a supposedly safe alternative, promised as being free of psychotropic components or their side-effects ^4^. With the exception of the treatment of Dravet’s syndrome, there is little clinical data on the efficacy and safety of CBD, particularly in the treatment of cancer ^5, 6^. Cannabidiol is currently approved in the European Union (EU) in a single medicinal product, namely Epidiolex® for the treatment of seizures in patients with two rare, severe forms of childhood-onset epilepsy. Apart from that, extemporaneous preparations in pharmacies are legally available on prescription in Germany and some other countries. However, most of the CBD products worldwide are available as food supplements or additives in food.

Commercial CBD products are usually crude extracts from whole hemp plants (i.e., including flowers and stems). In other ways (e.g., in extracting the food-approved plant parts such as seeds), contents in the range of 1–10% CBD, which are typically advertised, cannot be achieved. Also, the limited available literature and manufacturer data confirm that CBD products are usually extracted by supercritical CO _2_ or with solvents such as ethanol or isopropanol from the entire hemp plant ^6, 7^. Probably due to cost reasons for some products, no further specific enrichment or purification of CBD is conducted, so that the commercial extracts are regularly a cannabinoid mixture rather than pure CBD. Otherwise, extracts may be cleaned with different processes such as winterization, or partial fractionation using supercritical CO _2_. These extracts, which are typically called “full spectrum extracts” in difference to chemically pure CBD, are then mixed into ordinary edible oils such as sunflower oil, olive oil or hemp seed oil to obtain the so-called CBD oil ^6^.

The strategy to market CBD products as food supplements within the framework of food regulations seems to be the most common approach of CBD sellers. The most prevalent food supplement products are CBD oils in liquid form or CBD oil or hemp extract containing capsules. Some other products, derived from hemp extracts, are CBD chewing gum, and cannabis resin, wax or pollen products, while so-called “CBD flowers” are typically sold as plant material to prepare a tea-like infusion.

However, no significant food consumption of hemp extracts or hemp flowers containing CBD has been documented before 15 May 1997. These products are therefore classified as “novel” in the Novel Food catalogue of the European Commission under the entry “cannabinoids” and therefore require approval according to the Novel Food Regulation. Up to date (as of August 2020), no approved application is recorded. Basically, all available CBD products based on hemp extract marketed as food or food supplement within the EU are therefore illegally sold ^2^. To circumvent the strict safety requirements for medicinal or food products, some CBD products may be sold as other product categories (e.g., cosmetics, veterinary supplements, waxes, air fresheners or room fragrances), but the off-label use, human consumption, is clearly intended.

Despite the enforcement efforts of the food and medicinal product control authorities (e.g. the EU’s rapid alert system for food and feed (RASFF) lists over 120 alerts for CBD since 2018), a multitude of CBD products is available over the internet and in some retail stores, so that CBD is currently easily available to consumers.

Anecdotal cases ranging from indisposition to ∆ ^9^-THC-like effects have been reported to our institute from food control authorities in the German Federal State of Baden-Württemberg in the context of consumer complaint cases regarding CBD products. Some case reports of side effects of CBD products were published ^8, 9^, and a survey of 135 CBD users in the USA detected a high prevalence of side effects (30% dry mouth, 22% feeling high, 20% change in appetite, 19% fatigue) ^10^. Additionally, some pediatric studies in epilepsy patients with orally administered CBD also reported adverse effects such as drowsiness and fatigue that could be explained by pharmacological properties of ∆ ^9^-THC rather than of CBD ^11– 13^. Diarrhoea was an adverse outcome associated with CBD treatment in a meta-analysis of randomized clinical trials, after excluding studies of childhood epilepsy ^14^. Post marketing safety surveillance of a full spectrum hemp extract reported gastrointestinal symptoms as most common adverse event, however, they were infrequent (0.03%) ^15^.

Currently there are three hypotheses for the cause of the side effects: (i) a direct pharmacological effect of CBD, (ii) the degradation of CBD to ∆ ^9^-THC due to acidic hydrolysis in the stomach following oral consumption, and (iii) ∆ ^9^-THC directly contained in the products as by-product due to co-extraction and enrichment or contamination. In this article, the hypotheses are investigated including new evidence from original data.

## Methods

### CBD degradation

To investigate CBD degradation into ∆ ^9^-THC under acidic conditions, differently concentrated CBD in methanolic solutions was used in a range corresponding to typical amounts consumed with supplements based on commercial CBD (Supelco Cerilliant, certified reference material, #C-045, 1.0 mg/mL in methanol) supplied by Merck (Darmstadt, Germany). These solutions were exposed to an artificial gastric juice as well as different incubation times and stress factors such as storage under light and heat (see Table 1 for full experimental design). The solutions were stored either in standard freezer (-18°C) or refrigerator (8°C) or at room temperature (20°C). Increased temperatures were achieved using a thermostatically controlled laboratory drying oven type “UT6120” (Heraeus, Langenselbold, Germany) set to either 37°C or 60°C. The daylight condition was achieved by storage at a window (south side). For ultraviolet light exposure, six 25 W ultraviolet (UV) fluorescent tubes type “excellent E” (99.1% UVA) built into a facial tanner type “NT 446 U” (Dr. Kern GmbH, Mademühlen, Germany) were placed 15 cm from the surface of the solutions (open sample vials). In deviation of an experimental protocol of Merrick *et al*. ^17^, a gastric juice without addition of surfactants was used, which was strictly produced according to the European pharmacopoeia ^18^ (0.020 g NaCl + 0.032 g pepsin + 0.8 mL HCl (1 mol/L), filled up to 10 mL with water). As pure CBD was available only in methanolic solution, the final experimental setups contained 0.08 mol/L HCl and 1% methanol due to dilution (methanol residues in this order of magnitude are not interfering with the analysis).

**Table 1.** Cannabidiol (CBD) stability experiments under various storage conditions.

Experiment	Temperature (°C)	Light exposure	Storage time	Storage medium	CBD concentration in medium (μg/L)	Δ ^9^-THC formation ^1^
Negative control	-18	None	14 days	Methanol	1000	0%
Light	20	None	3 days	Methanol	1000	0%
	20	None	14 days	Methanol	1000	0%
	20	Daylight	3 days	Methanol	1000	0%
	20	Daylight	14 days	Methanol	1000	0%
	20	UVA	1 h	Methanol	1000	0%
	20	UVA	3 h	Methanol	1000	0%
Temperature	20	None	5 days	Methanol	1000	0%
	20	None	14 days	Methanol	1000	0%
	8	None	5 days	Methanol	1000	0%
	8	None	14 days	Methanol	1000	0%
	37	None	3 h	Methanol	1000	0%
	60	None	1 h	Methanol	1000	0%
Simulated gastric juice	37	None	1 h	Simulated gastric juice	200	0%
	37	None	2 h	Simulated gastric juice	200	0%
	37	None	3 h	Simulated gastric juice	200	0%
	37	None	1 h	Simulated gastric juice	400	0%
	37	None	2 h	Simulated gastric juice	400	0%
	37	None	3 h	Simulated gastric juice	400	0%
Positive control	20	None	14 days	Methanol / 1 mol/L HCl (50:50)	500	27%

^1^ Average of LC-MS/MS and UPLC-QTOF measurements (n=2) (for raw results see dataset ^16^, table sheet 1). Δ ^9^-THC formation calculated as % in relation to original CBD content.

Abbreviations: CBD: cannabidiol; Δ ^9^-THC: Δ ^9^-tetrahydrocannabinol; UVA: ultraviolet A; LC-MS/MS: liquid chromatography/tandem mass spectrometry; UPLC-QTOF: ultra-high pressure liquid chromatography/quadrupole time-of-flight mass spectrometry

To ensure the utmost analytical validity, all samples were independently measured on two different instruments, using a triple quadrupole mass spectrometer (TSQ Vantage, Thermo Fisher Scientific, San Jose, CA, USA) coupled with an LC system (1100 series, Agilent, Waldbronn, Germany) and also using a quadrupole time-of-flight (QTOF) mass spectrometer (X500, Sciex, Darmstadt, Germany) coupled with an UPLC system (1290 series, Agilent, Waldbronn, Germany). Both systems used the same type of separation column (Luna Omega Polar C18, 150 × 2.1 mm, 1.6 μm, 100 Å, Phenomenex, Aschaffenburg, Germany). The separation was isocratic with 25 % water (0.1 % formic acid) and 75 % acetonitrile (0.1 % formic acid) and a flow of 0.3 mL/min. In case of QTOF with 35 % water (0.1 % formic acid) and 65 % acetonitrile (0.1 % formic acid) and a flow of 0.45 mL/min. The evaluation took place after fragmentation of the mother ion into three mass traces for each compound. As quantifier for ∆ ^9^-THC and CBD, the mass transition m/z 315 to 193 was used. In case of QTOF, quantification was conducted over accurate mass and control of fragmentation pattern. CBD eluted as one of the first cannabinoids, a few minutes before ∆ ^9^-THC. As internal standards ∆ ^9^-THC-d _3_ (Supelco Cerilliant #T-011, 1.0 mg/mL in methanol) was used for the quantification of ∆ ^9^-THC (Supelco Cerilliant #T-005, 1.0 mg/mL in methanol), and cannabidiol-d _3_ (Supelco Cerilliant #C-084, 100 μg/mL in methanol) for quantification of CBD (Supelco Cerilliant #C-045, 1.0 mg/mL in methanol). The certified reference materials were obtained as solutions in ampoules of 1 mL, all supplied by Merck (Darmstadt, Germany). A limit of detection (LOD) of 5 ng/mL was determined. For both procedures, relative standard deviations better than 5% were achieved. Both methods are able to chromatographically separate ∆ ^9^-THC and CBD from their acids. Specificity was ensured using a certified reference material as a reference standard of THCA (Supelco Cerilliant #T-093, 1.0 mg/mL in acetonitrile). Baseline separation was achieved between ∆ ^9^-THC, ∆ ^8^-THC and THCA. Therefore, the reported values in this study are specific for ∆ ^9^-THC and CBD. In contrast to some previous studies based on gas chromatography, we do not report “total THC” or “total CBD”, which would be a sum of the free form and its acid.

### ∆ ^9^-THC contamination of commercial products

To study the possible influence of natively contained ∆ ^9^-THC in hemp products as a cause for side effects, a sampling of all available CBD products registered as food supplement in the German State Baden-Württemberg, other available hemp extract products in retail, as well as all products available at the warehouse of a large internet retailer were sampled between December 2018 and December 2019. A total of 67 samples (see Table 2 for product designations) were analysed using the above described liquid chromatographic method with tandem mass spectrometry (LC-MS/MS) for ∆ ^9^-THC content. For toxicological evaluation of the results, the lowest observed adverse effect level (LOAEL) of 2.5 mg ∆ ^9^-THC per day published by the European food safety authority (EFSA) based on human data (central nervous system effects and pulse increase) was used ^20^. Taking safety factors (factor 3 for extrapolation from LOAEL to no observed adverse effect level (NOAEL) and factor 10 for interindividual differences, total factor 30) into account, an acute reference dose (ARfD) of 1 μg ∆ ^9^-THC per kg body weight was derived ^20^. In their assessment, the Panel on Contaminants in the Food Chain of EFSA also considered interaction between ∆ ^9^-THC and CBD, but found the information controversial and not consistently antagonistic ^20 ^. This is consistent with more recent research of Solowij *et al.* ^21^ that the effects of ∆ ^9^-THC may even be enhanced by low-dose CBD (e.g., as found in food supplements) and may be particular prominent in infrequent cannabis users. However, the current scientific evidence does not allow for considering cumulative effects. The applicability of the acute reference dose (ARfD) of 1 μg ∆ ^9^-THC per kg body weight was re-confirmed by EFSA in 2020 ^22^. For further details on interpretation of results and toxicity assessment, see Lachenmeier *et al.* ^2^.

**Table 2.** Results of THC analysis in commercial hemp-based products from the German market (2018–2019).

Sample ID	Product	CBD [mg/day] (recommended daily dose according to labelling)	CBD [mg/day] (analysis) ^1^	Δ ^9^-THC [mg/day] (analysis) ^1^	Toxicity assessment according to Ref. 2
190267605	CBD oil	2000 ^2^	3140	30	THC > LOAEL
180630663	CBD oil supplement	200	- ^3^	9	THC > LOAEL
190595270	Hemp tea with flowers	- ^4^	- ^3^	5	THC > LOAEL
180776480	CBD oil supplement	74	51	4	THC > LOAEL
190490183	Hemp tea with flowers	- ^4^	19	4	THC > LOAEL
190595273	Hemp tea with flowers	- ^4^	- ^3^	3.6	THC > LOAEL
190595267	Hemp tea with flowers	- ^4^	16	3.3	THC > LOAEL
190203194	CBD pollen	- ^4^	- ^3^	2.6	THC > LOAEL
180598182	CBD hemp flower supplement	500	- ^3^	(2.3) ^5^	THC > LOAEL
190495001	Hemp tea with flowers	(3.8 % CBD/ package)	- ^3^	(2.3) ^5^	THC > LOAEL
190203193	CBD wax	660	860	(1.7) ^5^	THC > LOAEL
180781746	CBD chewing gum	15	30	(1.5) ^5^	THC > LOAEL
190400870	Hemp tea with flowers	"high CBD content"	16	(1.4) ^5^	THC > LOAEL
180198245	CBD buds (hemp flowers & leaves)	- ^4^	- ^3^	(1.3) ^5^	THC > LOAEL
180198246	CBD buds (hemp flowers & leaves)	- ^4^	- ^3^	(1.3) ^5^	THC > LOAEL
180598187	CBD hemp flower supplement	250	- ^3^	(1.3) ^5^	THC > LOAEL
190176314	Hemp tea with leaves and flowers	50	9	(0.5) ^5^	THC > LOAEL
190141197	CBD oil supplement	22.32	- ^3^	1.6	ARfD < THC < LOAEL
190203191	Supplement with hemp extract	- ^4^	- ^3^	0.7	ARfD < THC < LOAEL
190698985	CBD oil supplement	40	- ^3^	0.6	ARfD < THC < LOAEL
190400871	Hemp tea with flowers	- ^4^	8	0.6	ARfD < THC < LOAEL
190199739	Supplement with hemp extract	- ^4^	34	0.5	ARfD < THC < LOAEL
190660814	CBD oil supplement	30	- ^3^	0.5	ARfD < THC < LOAEL
190207787	CBD oil supplement	67.5	95	0.4	ARfD < THC < LOAEL
190332551	CBD oil supplement	42	- ^3^	0.3	ARfD < THC < LOAEL
190332552	CBD oil supplement	84	- ^3^	0.3	ARfD < THC < LOAEL
190332553	CBD oil supplement	166	- ^3^	0.3	ARfD < THC < LOAEL
190540832	Supplement with hemp extract	- ^4^	- ^3^	0.3	ARfD < THC < LOAEL
180565755	CBD oil supplement	24	18	0.2	ARfD < THC < LOAEL
180565756	CBD oil supplement	12	9	0.2	ARfD < THC < LOAEL
190203189	Supplement with hemp extract	- ^4^	- ^3^	0.2	ARfD < THC < LOAEL
190480260	Supplement with hemp juice powder	- ^4^	- ^3^	0.2	ARfD < THC < LOAEL
190180559	CBD wax	700	- ^3^	0.2	ARfD < THC < LOAEL
190480266	Hemp tea with leaves	- ^4^	- ^3^	0.2	ARfD < THC < LOAEL
190394018	CBD oil supplement	2000	- ^3^	0.2	ARfD < THC < LOAEL
190351382	CBD oil supplement	24	- ^3^	0.2	ARfD < THC < LOAEL
190480263	Supplement with hemp extract	- ^4^	- ^3^	0.2	ARfD < THC < LOAEL
190595265	Syrup with hemp flower extract	- ^4^	- ^3^	0.2	ARfD < THC < LOAEL
190080916	Supplement with hemp extract	- ^4^	- ^3^	0.1	ARfD < THC < LOAEL
190080917	Supplement with hemp extract	- ^4^	4	0.1	ARfD < THC < LOAEL
190303096	CBD chewing gum	5	- ^3^	0.1	ARfD < THC < LOAEL
190304229	CBD chewing gum	5	- ^3^	0.1	ARfD < THC < LOAEL
190696141	CBD oil supplement	7	- ^3^	0.1	ARfD < THC < LOAEL
190689579	CBD oil supplement	24	- ^3^	0.1	ARfD < THC < LOAEL
190480151	Supplement with hemp juice powder	- ^4^	- ^3^	0.1	ARfD < THC < LOAEL
190578889	Hemp seed with leaves (tea)	- ^4^	- ^3^	0.1	ARfD < THC < LOAEL
190203192	Supplement with hemp extract	- ^4^	- ^3^	0.07	THC > German guideline ^6^ THC < ARfD
190639434	CBD oil supplement	50	- ^3^	0.07	THC > German guideline ^6^ THC < ARfD
190639431	CBD oil supplement	38	- ^3^	0.07	THC > German guideline ^6^ THC < ARfD
190304228	CBD supplement	20	- ^3^	0.05	THC > German guideline ^6^ THC < ARfD
190468594	CBD oil supplement	4	- ^3^	0.05	THC > German guideline ^6^ THC < ARfD
190626611	Supplement with hemp juice powder	- ^4^	- ^3^	0.05	THC > German guideline ^6^ THC < ARfD
190626620	Supplement with hemp juice powder	- ^4^	- ^3^	0.04	THC > German guideline ^6^ THC < ARfD
190629508	CBD oil supplement	18	- ^3^	0.03	THC > German guideline ^6^ THC < ARfD
190629507	Supplement with hemp extract	12	- ^3^	0.02	THC > German guideline ^6^ THC < ARfD
190348163	Supplement with hemp extract	2	- ^3^	0.02	THC > German guideline ^6^ THC < ARfD
190272024	CBD oil	27	38	0.01	THC > German guideline ^6^ THC < ARfD
190601859	Supplement with hemp extract	100	- ^3^	0.01	THC > German guideline ^6^ THC < ARfD
190387558	CBD supplement	10	- ^3^	0.01	THC > German guideline ^6^ THC < ARfD
190664273	Cannabis shot (one portion)	- ^4^	- ^3^	0.008	THC > German guideline ^6^ THC < ARfD
190387560	CBD supplement	5	- ^3^	0.006	THC > German guideline ^6^ THC < ARfD
190378411	CBD Hemp Bears	20	- ^3^	0.004	THC > German guideline ^6^ THC < ARfD
190672010	CBD oil supplement	14	- ^3^	0.002	THC > German guideline ^6^ THC < ARfD
190387553	CBD supplement	5	- ^3^	0.002	THC > German guideline ^6^ THC < ARfD
190203186	Supplement with hemp extract	- ^4^	- ^3^	Not detectable	-
190387556	CBD supplement	4	- ^3^	Not detectable	-
190539777	CBD Lollipop	- ^4^	- ^3^	Not detectable	-

^1^ Average of 1–8 replicates measured with LC-MS/MS reported (for raw results see dataset
^16^, table sheet 2). Data reported for chromatographically separated CBD and Δ
^9^-THC, not including their acids.

^2^ No labelling about dosage provided on the label. For this reason, the consumption of the whole bottle at once was assumed as worst-case exposure scenario. Because the product was only labelled as “oil” and not as “food supplement”, this scenario is not deemed unrealistic, specifically since CBD is a novelty on the market and the product may be mistaken for a conventional edible oil.

^3^ Not analysed or outside calibration (most sample dilutions made for Δ
^9^-THC analysis by far exceed the linear range for CBD, so that a separate dilution would have to be made to obtain a valid result, which was not possible in the context of the current study).

^4^ No labelling provided by manufacturer.

^5^ Values in brackets mean that the LOAEL is not directly exceeded based on the recommended daily dose according to labelling, but may be exceeded in realistic exposure scenarios. For example, Δ
^9^-THC (mg/day) is calculated for food supplements on the basis of the recommended daily maximum dose or for 1 portion (if labelling of maximum recommended daily dose is missing). The LOAEL for these products may be exceeded with a probable intake of 2 portions/day. For tea products, a daily consumption of 8 g has been assumed if no other labelling was provided. However, much higher tea consumption is possible, so that a worst-case scenario has to be considered. For example, the very small portion size of 2.5 g labelled on the product with sample ID 190176314, would lead to a Δ
^9^-THC intake of 0.5 mg per day. However, if only 5 times this amount is consumed, which is neither unexpected nor impossible considering typically herbal tea consumption, the LOAEL may be exceeded. For all products, a case-by-case judgement was conducted, also considering manufacturers’ warning labels drawing attention to not exceeding the recommended daily intake.

^6^ The German guideline value for total THC (i.e. the sum of Δ
^9^-THC and Δ
^9^-tetrahydrocannabinolic acid (THCA)) content is 5 μg/kg in beverages, 5000 μg/kg in edible oils and 150 μg/kg in other food products (including food supplements)
^19^. Exceedance of the guideline value reported for Δ
^9^-THC alone without consideration of THCA.

Abbreviations: CBD: cannabidiol; THC: Δ
^9^-tetrahydrocannabinol; ARfD: acute reference dose of 1 μg THC per kg body weight
^20^; LOAEL: lowest observed adverse effect level of 2.5 mg Δ
^9^-THC per day
^20^; LC-MS/MS: liquid chromatography/tandem mass spectrometry

## Results and discussion

### Direct pharmacological effect of CBD as explanation of side effects

There is not much evidence to assume that chemically pure CBD may exhibit ∆ ^9^-THC-like side-effects. The World Health Organization (WHO) judged the compound as being well tolerated with a good safety profile ^3^. CBD doses in the food supplements on the market are typically much lower than the ones tested in clinical studies. Additionally, there is a 90-day experiment in rats with a hemp extract (consisting of 26% cannabinoids, out of which 96% were CBD and less than 1% ∆ ^9^-THC) from which a NOAEL of 100 mg/kg bw/day could be derived ^23^. Based on 100 mg/kg bw/day × 26% × 96%, this would be about 25 mg/kg bw/day for CBD (or 1750 mg/day for a person with a body weight of 70 kg). This NOAEL would not typically be reached by the CBD dosages in food supplements.

### CBD conversion into THC as explanation of side effects

Some, partly older, *in vitro* studies put up hypotheses about the conversion of CBD to ∆ ^9^-THC under acidic conditions such as in artificial gastric juice ^17, 24– 26^. If these proposals could be confirmed with *in vivo* data, consumers taking CBD orally could be exposed to such high ∆ ^9^-THC levels that the threshold for pharmacological action could be exceeded ^27^. However, taking a closer look at these *in vitro* studies raises some doubts. If CBD was to be converted to ∆ ^9^-THC in the stomach, typical ∆ ^9^-THC metabolites should be detectable in blood and urine, but this has not been observed in oral CBD studies ^28, 29^. Due to the contradicting results, a replication of the *in vitro* study of Merrick *et al*. ^17^ was conducted using an extended experimental design. A more selective LC-MS/MS method and also an ultra-high pressure liquid chromatographic method with quadrupole time-of-flight mass spectrometry (UPLC-QTOF) were used to investigate the CBD degradation.

Under these conditions in contrast to Merrick *et al*. ^17^, no conversion of CBD to ∆ ^9^-THC was observed in any of the samples. Only in case of the positive control (2 week storage in 0.5 mol/L HCl and 50% methanol), a complete degradation of CBD into 27% ∆ ^9^-THC and other not identified products (with fragments similar to the ones found in cannabinol and ∆ ^9^-THC fragmentations but with other retention times) was observed (Table 1, underlying data ^16^). From an analytical viewpoint, the use of less selective and specific analytical methods, especially from the point of chromatographic separation, could result in a situation in which certain CBD degradation products might easily be confused with ∆ ^9^-THC due to structural similarities. Thus, similar fragmentation patterns and potentially overlapping peaks under certain chromatographic conditions might have led to false positive results in the previous studies. In conclusion of our degradation experiments, we agree with more recent literature ^30, 31^ that CBD would not likely react to ∆ ^9^-THC under *in vivo* conditions. The only detectable influence leading to degradation is strong acidity, which should be avoided in CBD formulations to ensure stability of products ^32^.

### ∆ ^9^-THC contamination as cause of side effects

Out of 67 samples, 17 samples (25% of the collective) have the potential to exceed the ∆ ^9^-THC LOAEL and were assessed as harmful to health. 29 samples (43% of the collective) were classified as unsuitable for human consumption due to exceeding the ARfD (see Table 2, underlying data ^16^). Furthermore, all samples (100%) have been classified as non-compliant to Regulation (EU) 2015/2283 of the European Parliament and of the Council of 25 November 2015 on novel foods ^33^ and therefore being unauthorized novel foods ^34^. The labelling of all samples (100%) was also non-compliant to Regulation (EU) No 1169/2011 of the European Parliament and of the Council of 25 October 2011 on the provision of food information to consumers ^35^, e.g. due to lack of mandatory food information such as ingredients list or use of unapproved health claims in accordance to Regulation (EC) No 1924/2006 of the European Parliament and of the Council of 20 December 2006 on nutrition and health claims made on foods ^36^. In summary, none of the products in our survey was found as being fully compliant with European food regulations.

The ∆ ^9^-THC dose leading to intoxication is considered to be in the range of 10 to 20 mg (very high dose in heavy episodic cannabis users up to 60 mg) for cannabis smoking ^37^. The resorption of orally ingested ∆ ^9^-THC varies greatly inter-individually with respect to both total amount and resorption rate ^38^. This might be one of the reasons for the individually very different psychotropic effects. A single oral dose of 20 mg THC resulted in symptoms such as tachycardia, conjunctival irritation, “high sensation” or dysphoria in adults within one to four hours. In one in five adults, a single dose of 5 mg already showed corresponding symptoms ^39^.

Some of the CBD oil supplements contained ∆ ^9^-THC in doses up to 30 mg (in this case in the whole bottle of 10 ml), which can easily explain the adverse effects observed by some consumers. Most of the CBD oils with dosage of around 1 mg ∆ ^9^-THC per serving offer the possibility to achieve intoxicating and psychotropic effects due to this compound if the products are used off-label (i.e. increase of the labelled maximum recommended daily dose by factors of 3–5, which is probably not an unlikely scenario. Some manufacturers even suggest an increase of daily dosage over time). Generally, these products pose a risk to human health, especially in light of the German guideline value for total THC in these kind of products ^19, 40^. The German guideline value of 150 μg total THC/kg for foods in general including food supplements is several orders of magnitude below the actual contents of ∆ ^9^-THC in the CBD products, even without consideration of THCA.

Hence our results provide compelling evidence that THC natively contained in CBD products by contamination may be a direct cause for side effects of these products. Obviously, there is an involuntary or deliberate lack of quality control of CBD products. Claims of “THC-free”, used by most manufacturers, even of the highly contaminated products – sometimes based on unsuitable analytical methodologies with limits of detection in the percentage range –, have to be treated as fraudulent or deceptive food information.

## Conclusions

In light of the discussion about the three potential causative factors for side effects of CBD products, the described effects can be explained most probably by the presence of native THC as contaminant in the products rather than by direct action of CBD or its chemical transformation. The conclusions and findings of this study are further supported by the findings of Hazekamp ^6^ reporting data from the Netherlands on cannabis oils according to which the labelling information for CBD and ∆ ^9^-THC was often different from the actual contents. In 26 out of 46 products the ∆ ^9^-THC content was >1 g/100 mL. Further corresponding results were reported in a study from the USA, in which the CBD content was correctly declared for only 26 of 84 CBD products and 18 of the products had ∆ ^9^-THC contents >0.317 g/100 g ^41^.

CBD degradation products are currently unknown and need to be characterized and toxicologically assessed, e.g. within the context of the novel food registration process. Until then, the safety of the products remains questionable. Furthermore, standardization and purification of the extracts need to be improved and stability of commercial products during shelf life should be checked (e.g. to prevent CBD degradation by avoiding acidity in ingredients etc.).

In our opinion the systematically high ∆ ^9^-THC content of CBD products is clearly a “scandal” on the food market. Obviously, the manufacturers have – deliberately or in complete ignorance of the legal situation – placed unsafe and unapproved products on the market and thus exposed the consumer to an actually avoidable risk. In view of the growing market for such lifestyle food supplements, the effectiveness of the instrument of food business operators’ own responsibility for food safety must obviously be challenged.

It has been claimed by C. Hillard that “many CBD products would be delivering enough THC along with it to provide a bit of a high and that’s more likely where the relief is coming from” ^42^ and our results have partially corroborated this opinion for a substantial number of products on the German market. Similarly, a recent survey reported that 22% out of 135 users of CBD products reported “feeling high” as common side effect ^10^.

According to P. Pacher considering the situation in the USA, CBD users must be aware that they may be “participating in one of the largest uncontrolled clinical trials in history” ^42^. Currently we have no evidence that this claim is not also valid for the CBD market in the European Union, where obviously considerable numbers of unsafe and misleadingly labelled products are available. Due to consistent deficits in mandatory labelling including a lack of maximum recommended daily dose, dosages up to psychotropic levels (for THC) or pharmacological levels (for CBD) cannot be excluded with certainty. The risk also includes positive cannabis urine tests for several days, which may be expected from daily oral doses of more than 1 mg ∆ ^9^-THC ^1, 2, 43^. Therefore, more than 1/4 of products in our study would probably lead to false-positive urine tests, which could have grave consequences for persons occupationally or otherwise required to prove absence of drug use or of doping in professional sports ^44, 45^.

Obviously, the current regulatory framework is insufficient to adequately regulate products in the grey area between medicines and food supplements. For cannabis-derived products, such as CBD, the problem is aggravated by conflicting regulations in the narcotic, medicinal, and food law areas. For example, hemp extract-based products of similar composition could be treated as illegal narcotics, prescription-based medicinal products, or novel foods. According to press information, the EU commission is currently considering classifying cannabidiol products as narcotics, and hence as non-food products ^46^. Clearly for CBD products alongside other medicinal cannabis products, a regulated legalization (see e.g. Anderson *et al.* ^47^) would be preferable, introducing stricter regulations, such as mandatory labelling requirements, safety assessment, testing and pre-marketing approval (also see 29, 48).

## Data availability

### Underlying data

Open Science Framework: Dataset for “Are side effects of cannabidiol (CBD) products caused by delta9-tetrahydrocannabinol (THC) contamination?” (Version 2) https://doi.org/10.17605/OSF.IO/F7ZXY ^16^

This project contains the following underlying data:

- Dataset for 'Are side effects of cannabidiol (CBD) products caused by delta9-tetrahydrocannabinol (THC) contamination' F1000 Research.xlsx (Version 2) (Excel spreadsheet with data underlying Table 1 and Table 2, missing data/empty cells correspond to values outside calibration (CBD) or not measured)

Data are available under the terms of the Creative Commons Zero “No rights reserved” data waiver (CC0 1.0 Public domain dedication).

