# Are adverse effects of cannabidiol (CBD) products caused by tetrahydrocannabinol (THC) contamination?

**DOI:** 10.12688/f1000research.19931.4

**Published:** 2021-07-30

**Authors:** Dirk W. Lachenmeier, Stephanie Habel, Berit Fischer, Frauke Herbi, Yvonne Zerbe, Verena Bock, Tabata Rajcic de Rezende, Stephan G. Walch, Constanze Sproll

**Affiliations:** 1 Chemisches und Veterinäruntersuchungsamt (CVUA) Karlsruhe, Karlsruhe, 76187, Germany

**Keywords:** Tetrahydrocannabinol, cannabidiol, Cannabis sativa, hemp, food supplements, risk assessment, drug effects

## Abstract

Cannabidiol (CBD)-containing products are widely marketed as over the counter products, mostly as food supplements. Adverse effects reported in anecdotal consumer reports or during clinical studies were first assumed to be due to hydrolytic conversion of CBD to psychotropic Δ ^9^-tetrahydrocannabinol (Δ ^9^-THC) in the stomach after oral consumption. However, research of pure CBD solutions stored in simulated gastric juice or subjected to various storage conditions such as heat and light with specific liquid chromatographic/tandem mass spectrometric (LC/MS/MS) and ultra-high pressure liquid chromatographic/quadrupole time-of-flight mass spectrometric (UPLC-QTOF) analyses was unable to confirm THC formation. Another hypothesis for the  adverse effects of CBD products may be residual Δ ^9^-THC concentrations in the products as contamination, because most of them are based on hemp extracts containing the full spectrum of cannabinoids besides CBD. Analyses of 181 food products of the German market (mostly CBD oils) confirmed this hypothesis: 21 products (12%) contained Δ ^9^-THC above the lowest observed adverse effect level (2.5 mg/day). Inversely, CBD was present in the products below the no observed adverse effect level. Hence, it may be assumed that the adverse effects of some commercial CBD products are based on a low-dose effect of Δ ^9^-THC and not due to effects of CBD itself. The safety, efficacy and purity of commercial CBD products is highly questionable, and all of the products in our sample collection showed various non-conformities to European food law such as unsafe Δ ^9^-THC levels, hemp extracts or CBD isolates as non-approved novel food ingredients, non-approved health claims, and deficits in mandatory food labelling requirements. In view of the growing market for such lifestyle products, the effectiveness of the instrument of food business operators' own responsibility for product safety and regulatory compliance must obviously be challenged, and a strong regulatory framework for hemp products needs to be devised.

## Introduction

Since hemp has been re-approved for cultivation as an industrial crop in the form of low Δ ^9^-tetrahydrocannabinol (∆ ^9^-THC) hemp ( *Cannabis sativa* L.) varieties in the European Union (EU), components of the hemp plant are increasingly used for the production of foods and other consumer products such as liquids for electronic cigarettes ^1^.

From all hemp constituents, cannabidiol (CBD) is currently the compound with highest interest. In contrast to ∆ ^9^-THC, the major narcotic constituent of hemp, CBD is a non-psychotropic cannabinoid. It is currently being tested for its possible antispasmodic, anti-inflammatory, anxiolytic and antiemetic effects as a drug, e.g. for the treatment of epilepsy ^2, 3^. However, CBD products of all kinds can now also be purchased in organic shops, drug stores, specialised hemp and CBD stores, but also in supermarkets and via the Internet, often by advertising questionable “cure-all” properties including various unspecific health advantages. The marketing of CBD products is based on the current “hype” around medicinal hemp products, whereby the CBD products are offered as a supposedly safe alternative, promised as being free of psychotropic components or their adverse effects ^4^. With the exception of the treatment of Dravet’s syndrome, there is little clinical data on the efficacy and safety of CBD ^5, 6^. Cannabidiol is currently approved in the EU in a single medicinal product, namely Epidiolex® for the treatment of seizures in patients with two rare, severe forms of childhood-onset epilepsy. Apart from that, extemporaneous preparations in pharmacies are legally available on prescription in Germany and some other countries. However, most of the CBD products worldwide are available as food supplements or CBD-containing hemp extracts are used as ingredient in other foods.

Commercial CBD products are usually crude extracts from whole hemp plants (i.e., including flowers and stems). In other ways (e.g., in extracting the food-approved plant parts such as seeds), contents in the range of 1–10% CBD, which are typically advertised, cannot be achieved. Also, the limited available literature and manufacturer data confirm that CBD products are usually extracted by supercritical CO _2_ or with solvents such as ethanol or isopropanol from the entire hemp plant material, which typically has been decarboxylated before the process ^6, 7^. No further specific enrichment or purification of CBD is often conducted, so that the commercial extracts are regularly a cannabinoid mixture rather than pure CBD. Otherwise, extracts may be cleaned with different processes such as winterization, or partial fractionation using supercritical CO _2_. These extracts, which are typically called “full spectrum extracts” in difference to chemically pure CBD (such as isolated or synthesized CBD), are then mixed into ordinary edible oils such as sunflower oil, olive oil or hemp seed oil to obtain the so-called CBD oil ^6^.

The strategy to market CBD products as food supplements within the framework of food regulations seems to be the most common approach of CBD sellers. The most prevalent food supplement products are CBD oils in liquid form or hemp extract containing capsules. Some other products, derived from hemp extracts, are CBD chewing gum, and cannabis resin, wax or pollen products, while so-called “CBD flowers” are typically sold as plant material to prepare a tea-like infusion or as an herbal product for smoking.

However, no significant food consumption of CBD products has been documented before 15 May 1997. These products are therefore classified as “novel” in the Novel Food catalogue of the European Commission under the entry “cannabinoids” and therefore require approval according to the Novel Food Regulation. Up to date (as of July 2021), no approved application is documented while the approval process of five applications for synthetic CBD is currently in progress. Basically, all available CBD products based on hemp extract but also those based on isolated or synthesized CBD, which are marketed as food or food supplement within the EU, are therefore illegally sold ^2^. To circumvent the strict safety requirements for medicinal or food products, some CBD products may be sold as other product categories (e.g., cosmetics, veterinary supplements, waxes, flavourings, air fresheners or room fragrances), but the off-label use, human consumption, is clearly intended.

Despite the enforcement efforts of the food and medicinal product control authorities (e.g. the EU’s rapid alert system for food and feed (RASFF) lists over 160 alerts for CBD since 2018), a multitude of CBD products is available on the internet and in some retail stores, so that CBD is currently easily available to consumers.

Despite the lack of mandatory nutrivigilance in the EU, anecdotal cases ranging from indisposition to ∆ ^9^-THC-like effects have been reported to our institute from food control authorities in the German Federal State of Baden-Württemberg in the context of consumer complaint cases regarding CBD products. Several case reports of adverse effects of CBD products have also been published ^8– 11^, and a survey of 135 CBD users in the USA detected a high prevalence of adverse effects (30% dry mouth, 22% feeling high, 20% change in appetite, 19% fatigue) ^12^. Additionally, some paediatric studies in epilepsy patients with orally administered CBD also reported adverse effects such as drowsiness and fatigue that could be explained by pharmacological properties of ∆ ^9^-THC rather than of CBD ^13– 15^. Respiratory depression was reported in a case of CBD overdose in a paediatric patient ^16^.

Diarrhoea was an adverse outcome associated with CBD treatment in a meta-analysis of randomized clinical trials, after excluding studies of childhood epilepsy ^17^. Post marketing safety surveillance of a full spectrum hemp extract reported gastrointestinal symptoms as most common adverse effect, however, they were infrequent (0.03%) ^18^. More recently, the epidemiology of CBD-related cases in the national poison data system of the USA was assessed. Cannabidiol cases increased from 0% in 2009–2018 to 17% of all cases in 2019 ^19^. Among the exposures in which CBD was the only reported substance (n=1275), the most common symptoms were mild central nervous system depression (10%), tachycardia (6%), dizziness/vertigo (5%), vomiting (5%), nausea (5%) and agitation (4%) ^20^. 

Currently there are three hypotheses for the cause of the adverse effects: (i) a direct pharmacological effect of CBD, (ii) the degradation of CBD to ∆ ^9^-THC due to acidic hydrolysis in the stomach following oral consumption, and (iii) ∆ ^9^-THC directly contained in the products as by-product due to co-extraction and enrichment or contamination such as formation from CBD degradation during storage. In this article, the hypotheses are investigated including new evidence from original data.

## Methods

### CBD degradation

To investigate CBD degradation into ∆ ^9^-THC under acidic conditions, differently concentrated CBD in methanolic solutions was used in a range corresponding to typical amounts consumed with supplements based on commercial CBD (Supelco Cerilliant, certified reference material, #C-045, 1.0 mg/mL in methanol) supplied by Merck (Darmstadt, Germany). These solutions were exposed to an artificial gastric juice as well as different incubation times and stress factors such as storage under light and heat (see Table 1 for full experimental design). The solutions were stored either in standard freezer (-18°C) or refrigerator (8°C) or at room temperature (20°C). Increased temperatures were achieved using a thermostatically controlled laboratory drying oven type “UT6120” (Heraeus, Langenselbold, Germany) set to either 37°C or 60°C. The daylight condition was achieved by storage at a window (south side). For ultraviolet light exposure, six 25 W ultraviolet (UV) fluorescent tubes type “excellent E” (99.1% UVA) built into a facial tanner type “NT 446 U” (Dr. Kern GmbH, Mademühlen, Germany) were placed 15 cm from the surface of the solutions (open sample vials). In deviation of an experimental protocol of Merrick *et al*. ^21^, a gastric juice without addition of surfactants was used, which was strictly produced according to the European pharmacopoeia ^22^ (0.020 g NaCl + 0.032 g pepsin + 0.8 mL HCl (1 mol/L), filled up to 10 mL with water). As pure CBD was available only in methanolic solution, the final experimental setups contained 0.08 mol/L HCl and 1% methanol due to dilution (methanol residues in this order of magnitude are not interfering with the analysis).

**Table 1.** Cannabidiol (CBD) stability experiments under various storage conditions.

Experiment	Temperature (°C)	Light exposure	Storage time	Storage medium	CBD concentration in medium (μg/L)	Δ ^9^-THC formation ^1^
Negative control	-18	None	14 days	Methanol	1000	0%
Light	20	None	3 days	Methanol	1000	0%
	20	None	14 days	Methanol	1000	0%
	20	Daylight	3 days	Methanol	1000	0%
	20	Daylight	14 days	Methanol	1000	0%
	20	UVA	1 h	Methanol	1000	0%
	20	UVA	3 h	Methanol	1000	0%
Temperature	20	None	5 days	Methanol	1000	0%
	20	None	14 days	Methanol	1000	0%
	8	None	5 days	Methanol	1000	0%
	8	None	14 days	Methanol	1000	0%
	37	None	3 h	Methanol	1000	0%
	60	None	1 h	Methanol	1000	0%
Simulated gastric juice	37	None	1 h	Simulated gastric juice	200	0%
	37	None	2 h	Simulated gastric juice	200	0%
	37	None	3 h	Simulated gastric juice	200	0%
	37	None	1 h	Simulated gastric juice	400	0%
	37	None	2 h	Simulated gastric juice	400	0%
	37	None	3 h	Simulated gastric juice	400	0%
Positive control	20	None	14 days	Methanol / 1 mol/L HCl (50:50)	500	27%

^1^ Average of LC-MS/MS and UPLC-QTOF measurements (n=2) (for raw results see dataset ^23^, table sheet 1). Δ ^9^-THC formation calculated as % in relation to original CBD content.

Abbreviations: CBD: cannabidiol; Δ ^9^-THC: Δ ^9^-tetrahydrocannabinol; UVA: ultraviolet A; LC-MS/MS: liquid chromatography/tandem mass spectrometry; UPLC-QTOF: ultra-high pressure liquid chromatography/quadrupole time-of-flight mass spectrometry

To ensure the utmost analytical validity, all samples were independently measured on two different instruments, using a triple quadrupole mass spectrometer (TSQ Vantage, Thermo Fisher Scientific, San Jose, CA, USA) coupled with an LC system (1100 series, Agilent, Waldbronn, Germany) and also using a quadrupole time-of-flight (QTOF) mass spectrometer (X500, Sciex, Darmstadt, Germany) coupled with an UPLC system (1290 series, Agilent, Waldbronn, Germany). Both systems used the same type of separation column (Luna Omega Polar C18, 150 × 2.1 mm, 1.6 μm, 100 Å, Phenomenex, Aschaffenburg, Germany). The separation was isocratic with 25 % water (0.1 % formic acid) and 75 % acetonitrile (0.1 % formic acid) and a flow of 0.3 mL/min. In case of QTOF with 35 % water (0.1 % formic acid) and 65 % acetonitrile (0.1 % formic acid) and a flow of 0.45 mL/min. The evaluation took place after fragmentation of the mother ion into three mass traces for each compound. As quantifier for ∆ ^9^-THC and CBD, the mass transition m/z 315 to 193 was used. In case of QTOF, quantification was conducted over accurate mass and control of fragmentation pattern. CBD eluted as one of the first cannabinoids, a few minutes before ∆ ^9^-THC. As internal standards ∆ ^9^-THC-d _3_ (Supelco Cerilliant #T-011, 1.0 mg/mL in methanol) was used for the quantification of ∆ ^9^-THC (Supelco Cerilliant #T-005, 1.0 mg/mL in methanol), and cannabidiol-d _3_ (Supelco Cerilliant #C-084, 100 μg/mL in methanol) for quantification of CBD (Supelco Cerilliant #C-045, 1.0 mg/mL in methanol). The certified reference materials were obtained as solutions in ampoules of 1 mL, all supplied by Merck (Darmstadt, Germany). A limit of detection (LOD) of 5 ng/mL was determined. For both procedures, relative standard deviations better than 5% were achieved. Both methods are able to chromatographically separate ∆ ^9^-THC and CBD from their acids. Specificity was ensured using a certified reference material as a reference standard of THCA (Supelco Cerilliant #T-093, 1.0 mg/mL in acetonitrile). Baseline separation was achieved between ∆ ^9^-THC, ∆ ^8^-THC and THCA. Therefore, the reported values in this study are specific for ∆ ^9^-THC and CBD. In contrast to some previous studies based on gas chromatography, we do not report “total THC” or “total CBD”, which would be a sum of the free form and its acid.

### ∆ ^9^-THC contamination of commercial products

To study the possible influence of natively contained ∆ ^9^-THC in hemp products as a cause for adverse effects, a sampling of available CBD products registered as food supplement in the German State Baden-Württemberg, other available hemp extract products in retail, as well as all products available at the warehouse of a large internet retailer were sampled between December 2018 and December 2020. A total of 181 samples (see Table 2 for product designations) were analysed using the above-described liquid chromatographic method with tandem mass spectrometric detection (LC-MS/MS) for ∆ ^9^-THC content. For 2020 samples, the following parameters of the method were changed: separation column (Raptor, ARC-18, 150 × 2.1 mm, 2.7 μm, Restek, Bad Homburg, Germany). The separation was a gradient starting with 20% eluent A (0.1 % formic acid in water) and 80% eluent B (0.1 % formic acid in methanol) for 18 min, followed by 5% A and 95% B for 5 min, and back to 20% A and 80% B for 7 min. All methods were validated and externally accredited according to ISO 17025 standard. Recently, the method reported satisfactory results for ∆ ^9^-THC during the international government chemist CBD food and cosmetic ring rial ^28^.

**Table 2.** Results of THC analysis in commercial hemp-based products from the German market (2018–2020).

Sample ID	Product	CBD [mg/day] (recommended daily dose according to labelling)	CBD [mg/day] (analysis) ^1^	Δ ^9^-THC [mg/day] (analysis) ^1^	Toxicity assessment according to Ref. 2
190267605	CBD oil	2000 ^2^	3140	30	THC > LOAEL
200517333	Hemp CBD Oil	1500	- ^3^	15	THC > LOAEL
180630663	CBD oil supplement	200	- ^3^	9.1	THC > LOAEL
200536492	CBD oil	2400	- ^3^	6.7	THC > LOAEL
200055880	CBD liquid - ^4^	400	272	6.5	THC > LOAEL
200427032	Hemp seed oil	- ^5^	- ^3^	6.4	THC > LOAEL
190595270	Hemp tea with flowers	- ^5^	- ^3^	5.1	THC > LOAEL
190490183	Hemp tea with flowers	- ^5^	19	4.0	THC > LOAEL
180776480	CBD oil supplement	74	51	3.9	THC > LOAEL
190595273	Hemp tea with flowers	- ^5^	- ^3^	3.6	THC > LOAEL
190595267	Hemp tea with flowers	- ^5^	16	3.3	THC > LOAEL
190203194	CBD pollen	- ^5^	- ^3^	2.6	THC > LOAEL
190495001	Hemp tea with flowers	(3.8 % CBD/package)	- ^3^	(2.3) ^6^	THC > LOAEL
180598182	CBD hemp flower supplement	500	- ^3^	(2.3) ^6^	THC > LOAEL
190203193	CBD wax	660	860	(1.7) ^6^	THC > LOAEL
180781746	CBD chewing gum	15	30	(1.5) ^6^	THC > LOAEL
190400870	Hemp tea with flowers	"high CBD content"	16	(1.4) ^6^	THC > LOAEL
180198245	CBD buds (hemp flowers & leaves)	- ^5^	- ^3^	(1.3) ^6^	THC > LOAEL
180598187	CBD hemp flower supplement	250	- ^3^	(1.3) ^6^	THC > LOAEL
180198246	CBD buds (hemp flowers & leaves)	- ^5^	- ^3^	(1.3) ^6^	THC > LOAEL
190176314	Hemp tea with leaves and flowers	50	9	(0.5) ^6^	THC > LOAEL
200308438	Hemp tea with flowers	- ^5^	- ^3^	2.4	ARfD < THC < LOAEL
200498262	Hemp tea	- ^5^	- ^3^	2.3	ARfD < THC < LOAEL
200404608	CBD liquid - ^4^	500	- ^3^	1.7	ARfD < THC < LOAEL
190141197	CBD oil supplement	22.32	- ^3^	1.6	ARfD < THC < LOAEL
190722404	Hemp tea	- ^5^	3.8	1.3	ARfD < THC < LOAEL
200055883	CBD liquid- ^4^	300	256	1.2	ARfD < THC < LOAEL
200049885	CBD liquid- ^4^	300	229	1.0	ARfD < THC < LOAEL
200064492	CBD liquid- ^4^	100	73	0.9	ARfD < THC < LOAEL
200308470	CBD liquid- ^4^	- ^5^	- ^3^	0.8	ARfD < THC < LOAEL
200100780	Hemp tea with flowers	- ^5^	- ^3^	0.7	ARfD < THC < LOAEL
190203191	Supplement with hemp extract	- ^5^	- ^3^	0.7	ARfD < THC < LOAEL
200461203	CBD liquid- ^4^	- ^5^	- ^3^	0.7	ARfD < THC < LOAEL
200663956	CBD flowers	- ^5^	- ^3^	0.7	ARfD < THC < LOAEL
200308466	CBD liquid- ^4^	- ^5^	- ^3^	0.7	ARfD < THC < LOAEL
200663959	CBD flowers	- ^5^	- ^3^	0.7	ARfD < THC < LOAEL
190698985	CBD oil supplement	40	- ^3^	0.6	ARfD < THC < LOAEL
200663960	CBD flowers	- ^5^	- ^3^	0.6	ARfD < THC < LOAEL
190400871	Hemp tea with flowers	- ^5^	8	0.6	ARfD < THC < LOAEL
200663957	CBD flowers	- ^5^	- ^3^	0.6	ARfD < THC < LOAEL
200622669	Hemp tea with leaves	- ^5^	- ^3^	0.6	ARfD < THC < LOAEL
190199739	Supplement with hemp extract	- ^5^	34	0.5	ARfD < THC < LOAEL
190660814	CBD oil supplement	30	- ^3^	0.5	ARfD < THC < LOAEL
200064491	CBD liquid- ^4^	100	58	0.5	ARfD < THC < LOAEL
200100857	Hemp tea with flowers	- ^5^	5	0.5	ARfD < THC < LOAEL
200713361	CBD oil	31	- ^3^	0.5	ARfD < THC < LOAEL
200461204	CBD liquid- ^4^	- ^5^	- ^3^	0.5	ARfD < THC < LOAEL
200663958	CBD flowers	- ^5^	- ^3^	0.4	ARfD < THC < LOAEL
190810601	CBD liquid- ^4^	250	214	0.4	ARfD < THC < LOAEL
190207787	CBD oil supplement	67.5	95	0.4	ARfD < THC < LOAEL
190332551	CBD oil supplement	42	- ^3^	0.3	ARfD < THC < LOAEL
190332552	CBD oil supplement	84	- ^3^	0.3	ARfD < THC < LOAEL
200498263	Hemp tea	- ^5^	- ^3^	0.3	ARfD < THC < LOAEL
200669073	CBD oil	- ^5^	- ^3^	0.3	ARfD < THC < LOAEL
200663954	CBD flowers	- ^5^	- ^3^	0.3	ARfD < THC < LOAEL
200308440	CBD oil	- ^5^	- ^3^	0.3	ARfD < THC < LOAEL
190332553	CBD oil supplement	166	- ^3^	0.3	ARfD < THC < LOAEL
190540832	Supplement with hemp extract	- ^5^	- ^3^	0.3	ARfD < THC < LOAEL
200404603	CBD liquid- ^4^	1000	- ^3^	0.2	ARfD < THC < LOAEL
180565755	CBD oil supplement	24	18	0.2	ARfD < THC < LOAEL
190480260	Supplement with hemp juice powder	- ^5^	- ^3^	0.2	ARfD < THC < LOAEL
190203189	Supplement with hemp extract	- ^5^	- ^3^	0.2	ARfD < THC < LOAEL
190180559	CBD wax	700	- ^3^	0.2	ARfD < THC < LOAEL
190661483	CBD oil supplement	24	0.1	0.2	ARfD < THC < LOAEL
200308465	CBD liquid- ^4^	- ^5^	- ^3^	0.2	ARfD < THC < LOAEL
200586742	Herbal Tea	- ^5^	- ^3^	0.2	ARfD < THC < LOAEL
180565756	CBD oil supplement	12	9	0.2	ARfD < THC < LOAEL
190480266	Hemp tea with leaves	- ^5^	- ^3^	0.2	ARfD < THC < LOAEL
200308444	CBD oil	- ^5^	- ^3^	0.2	ARfD < THC < LOAEL
200308441	CBD oil	- ^5^	- ^3^	0.2	ARfD < THC < LOAEL
200663955	CBD flowers	- ^5^	- ^3^	0.2	ARfD < THC < LOAEL
190394018	CBD oil supplement	2000	- ^3^	0.2	ARfD < THC < LOAEL
190351382	CBD oil supplement	24	- ^3^	0.2	ARfD < THC < LOAEL
190480263	Supplement with hemp extract	- ^5^	- ^3^	0.2	ARfD < THC < LOAEL
190595265	Syrup with hemp flower extract	- ^5^	- ^3^	0.2	ARfD < THC < LOAEL
200064256	CBD liquid- ^4^	333	294	0.2	ARfD < THC < LOAEL
200308447	CBD oil	- ^5^	- ^3^	0.2	ARfD < THC < LOAEL
190080917	Supplement with hemp extract	- ^5^	4	0.1	ARfD < THC < LOAEL
200308442	CBD oil	- ^5^	- ^3^	0.1	ARfD < THC < LOAEL
200669072	CBD oil	- ^5^	- ^3^	0.1	ARfD < THC < LOAEL
210094426	CBD oil	- ^5^	- ^3^	0.1	ARfD < THC < LOAEL
200055882	CBD liquid- ^4^	100	45	0.1	ARfD < THC < LOAEL
200557132	Hemp tea	- ^5^	- ^3^	0.1	ARfD < THC < LOAEL
200068141	CBD liquid- ^4^	500	400	0.1	ARfD < THC < LOAEL
200308445	CBD oil	- ^5^	- ^3^	0.1	ARfD < THC < LOAEL
200055874	CBD liquid- ^4^	600	578	0.1	ARfD < THC < LOAEL
200541751	Tea with hemp leaves	- ^5^	- ^3^	0.1	ARfD < THC < LOAEL
190303096	CBD chewing gum	5	- ^3^	0.1	ARfD < THC < LOAEL
190696142	CBD oil	24	0.02	0.1	ARfD < THC < LOAEL
200498257	CBD oil	50	- ^3^	0.1	ARfD < THC < LOAEL
190696141	CBD oil supplement	7	- ^3^	0.1	ARfD < THC < LOAEL
200421839	CBD oil	8.8	- ^3^	0.1	ARfD < THC < LOAEL
200424875	CBD oil	8.8	- ^3^	0.1	ARfD < THC < LOAEL
190304229	CBD chewing gum	5	- ^3^	0.1	ARfD < THC < LOAEL
200498265	Hemp tea	- ^5^	- ^3^	0.1	ARfD < THC < LOAEL
200421841	CBD hemp extract supplement	16	- ^3^	0.1	ARfD < THC < LOAEL
200429640	CBD hemp extract supplement	16	- ^3^	0.1	ARfD < THC < LOAEL
200434661	CBD hemp extract supplement	16	- ^3^	0.1	ARfD < THC < LOAEL
200442385	CBD hemp extract supplement	16	- ^3^	0.1	ARfD < THC < LOAEL
190661482	CBD oil	24	0.09	0.1	ARfD < THC < LOAEL
190689579	CBD oil supplement	24	- ^3^	0.1	ARfD < THC < LOAEL
200406179	CBD oil	600	- ^3^	0.1	ARfD < THC < LOAEL
190080916	Supplement with hemp extract	- ^5^	- ^3^	0.1	ARfD < THC < LOAEL
200100859	CBD oil	47.5	- ^3^	0.09	THC < ARfD
200308450	CBD oil	- ^5^	- ^3^	0.09	THC < ARfD
200308443	CBD oil	- ^5^	- ^3^	0.09	THC < ARfD
200421840	CBD flavour oil	- ^5^	- ^3^	0.09	THC < ARfD
200049887	CBD liquid- ^4^	100	67	0.09	THC < ARfD
190480151	Supplement with hemp juice powder	- ^5^	- ^3^	0.08	THC < ARfD
200633636	CBD oil	11	- ^3^	0.08	THC < ARfD
200625847	CBD oil	24	- ^3^	0.08	THC < ARfD
200625849	CBD oil	18	- ^3^	0.08	THC < ARfD
200421842	CBD oil	12	- ^3^	0.08	THC < ARfD
200429639	CBD oil	12	- ^3^	0.08	THC < ARfD
200100781	Hemp extract	- ^5^	- ^3^	0.08	THC < ARfD
200633637	CBD oil	20	- ^3^	0.08	THC < ARfD
200699058	Hemp CBD supplement	30	- ^3^	0.07	THC < ARfD
190578889	Hemp seed with leaves (tea)	- ^5^	- ^3^	0.07	THC < ARfD
190639434	CBD oil supplement	50	- ^3^	0.07	THC < ARfD
200308449	CBD oil	- ^5^	- ^3^	0.07	THC < ARfD
190639431	CBD oil supplement	38	- ^3^	0.07	THC < ARfD
200405892	Hemp tea	- ^5^	- ^3^	0.07	THC < ARfD
190203192	Supplement with hemp extract	- ^5^	- ^3^	0.07	THC < ARfD
200633635	CBD oil	9	- ^3^	0.07	THC < ARfD
200040660	CBD liquid- ^4^	30	25	0.06	THC < ARfD
200308446	CBD oil	- ^5^	- ^3^	0.06	THC < ARfD
200308448	CBD oil	- ^5^	- ^3^	0.06	THC < ARfD
200092586	Hemp tea	- ^5^	- ^3^	0.06	THC < ARfD
200055034	CBD liquid- ^4^	120	95	0.06	THC < ARfD
200404607	CBD liquid- ^4^	1000	- ^3^	0.06	THC < ARfD
200622690	Supplements with hemp	- ^5^	- ^3^	0.05	THC < ARfD
190304228	CBD supplement	20	- ^3^	0.05	THC < ARfD
190468594	CBD oil supplement	4	- ^3^	0.05	THC < ARfD
190626611	Supplement with hemp juice powder	- ^5^	- ^3^	0.05	THC < ARfD
200530868	CBD oil	- ^5^	- ^3^	0.04	THC < ARfD
190626620	Supplement with hemp juice powder	- ^5^	- ^3^	0.04	THC < ARfD
200626107	CBD oil	1.5	- ^3^	0.04	THC < ARfD
200626105	CBD oil	2.4	- ^3^	0.04	THC < ARfD
200068143	CBD liquid- ^4^	100	62	0.04	THC < ARfD
200429638	CBD flavour oil	15.5	- ^3^	0.03	THC < ARfD
200530872	CBD oil	- ^5^	- ^3^	0.03	THC < ARfD
200666376	CBD oil	50	- ^3^	0.03	THC < ARfD
200068142	CBD liquid- ^4^	100	57	0.03	THC < ARfD
190629508	CBD oil supplement	18	- ^3^	0.03	THC < ARfD
200699054	CBD oil	12.6	- ^3^	0.03	THC < ARfD
200578707	CBD oil	6.5	- ^3^	0.03	THC < ARfD
200055873	CBD liquid- ^4^	500	50	0.02	THC < ARfD
200699051	CBD oil	50	- ^3^	0.02	THC < ARfD
200582816	Hemp Oil	- ^5^	- ^3^	0.02	THC < ARfD
190629507	Supplement with hemp extract	12	- ^3^	0.02	THC < ARfD
200530867	CBD oil	- ^5^	- ^3^	0.02	THC < ARfD
200100856	CBD oil	- ^5^	- ^3^	0.02	THC < ARfD
200055032	CBD liquid- ^4^	30	20	0.02	THC < ARfD
200626089	CBD oil	29	- ^3^	0.02	THC < ARfD
200530865	CBD oil	- ^5^	- ^3^	0.02	THC < ARfD
200622701	Supplements with hemp	18	- ^3^	0.02	THC < ARfD
190348163	Supplement with hemp extract	2	- ^3^	0.02	THC < ARfD
200626099	CBD oil	15	- ^3^	0.02	THC < ARfD
200049883	CBD liquid- ^4^	100	55	0.01	THC < ARfD
190601859	Supplement with hemp extract	100	- ^3^	0.01	THC < ARfD
190272024	CBD oil	27	38	0.01	THC < ARfD
190387558	CBD supplement	10	- ^3^	0.01	THC < ARfD
190810624	CBD liquid- ^4^	100	61	0.009	THC < ARfD
200117019	Hemp juice shot	- ^5^	0.2	0.008	THC < ARfD
190664273	Cannabis shot (one portion)	- ^5^	- ^3^	0.008	THC < ARfD
200055056	CBD liquid- ^4^	30	17	0.008	THC < ARfD
200626101	CBD oil	7.5	- ^3^	0.008	THC < ARfD
200064257	CBD liquid- ^4^	30	13	0.007	THC < ARfD
200626102	CBD oil	8.5	- ^3^	0.006	THC < ARfD
200626097	CBD oil	30	- ^3^	0.006	THC < ARfD
190387560	CBD supplement	5	- ^3^	0.006	THC < ARfD
200626094	CBD oil	38	- ^3^	0.005	THC < ARfD
190378411	CBD Hemp Bears	20	- ^3^	0.004	THC < ARfD
200622667	Supplements with hemp	- ^5^	0.09	0.004	THC < ARfD
190672010	CBD oil supplement	14	- ^3^	0.002	THC < ARfD
190387553	CBD supplement	5	- ^3^	0.002	THC < ARfD
200541745	CBD flavour oil	32	- ^3^	0.002	THC < ARfD
200541750	CBD flavour oil	32	- ^3^	0.001	THC < ARfD
190203186	Supplement with hemp extract	- ^5^	- ^3^	n.d.	-
190387556	CBD supplement	4	- ^3^	n.d.	-
190539777	CBD Lollipop	- ^5^	- ^3^	n.d.	-

^1^ Average of 1–9 replicates measured with LC-MS/MS reported (for raw results see dataset ^23^, table sheet 2). Data reported for chromatographically separated CBD and Δ ^9^-THC, not including their acids. ^2^ No labelling about dosage provided on the label. For this reason, the consumption of the whole bottle at once was assumed as worst-case exposure scenario. Because the product was only labelled as “oil” and not as “food supplement”, this scenario is not deemed unrealistic, specifically since CBD is a novelty on the market and the product may be mistaken for a conventional edible oil ^24^. ^3^ Not analysed or outside calibration (most sample dilutions made for Δ ^9^-THC analysis by far exceed the linear range for CBD, so that a separate dilution would have to be made to obtain a valid result, which was not possible in the context of the current study). ^4^ CBD liquid intended to refill electronic cigarettes. No national or international guideline for risk assessment of THC in liquids is available. For risk assessment, a LOAEL for inhalation of 2.8 mg/day was assumed ^25^, which was extrapolated to an ARfD of 1 µg/kg body weight using a safety factor of 30 and a bodyweight of 70 kg (also see rationale for oral ARfD in the text). An exposure of 10 ml liquid per day was assumed considering worst case exposure to vaping liquids ranging between 5–25 ml/day ^26^. ^5^ No labelling provided by manufacturer. ^6^ Values in brackets mean that the LOAEL is not directly exceeded based on the recommended daily dose according to labelling, but may be exceeded in realistic exposure scenarios. For example, Δ ^9^-THC (mg/day) is calculated for food supplements on the basis of the recommended daily maximum dose or for 1 portion (if labelling of maximum recommended daily dose is missing). The LOAEL for these products may be exceeded with a probable intake of 2 portions/day. For tea products, a daily consumption of 8 g has been assumed if no other labelling was provided. However, much higher tea consumption is possible, so that a worst-case scenario has to be considered. For example, the very small portion size of 2.5 g labelled on the product with sample ID 190176314, would lead to a Δ ^9^-THC intake of 0.5 mg per day. However, if only 5 times this amount is consumed, which is neither unexpected nor impossible considering typical herbal tea consumption, the LOAEL may be exceeded. For all products, a case-by-case judgement was conducted, also considering manufacturers’ warning labels drawing attention to not exceeding the recommended daily intake. Also see Lachenmeier and Walch for further details on estimation of daily doses ^24^. Abbreviations: CBD: cannabidiol; THC: Δ ^9^-tetrahydrocannabinol; ARfD: acute reference dose of 1 μg THC per kg body weight ^27^; LOAEL: lowest observed adverse effect level of 2.5 mg Δ ^9^-THC per day ^27^; LC-MS/MS: liquid chromatography/tandem mass spectrometry; n.d.: not detectable

For toxicological evaluation of the results, the lowest observed adverse effect level (LOAEL) of 2.5 mg ∆ ^9^-THC per day published by the European food safety authority (EFSA) based on human data (central nervous system effects and pulse increase) was used ^27^. Taking safety factors (factor 3 for extrapolation from LOAEL to no observed adverse effect level (NOAEL) and factor 10 for interindividual differences, total factor 30) into account, an acute reference dose (ARfD) of 1 μg ∆ ^9^-THC per kg body weight was derived ^27^. In their assessment, the Panel on Contaminants in the Food Chain of EFSA also considered interaction between ∆ ^9^-THC and CBD, but found the information controversial and not consistently antagonistic ^27^. This is consistent with more recent research of Solowij *et al.* ^29^ that the effects of ∆ ^9^-THC may even be enhanced by low-dose CBD (e.g., as found in food supplements) and may be particular prominent in infrequent cannabis users. However, the current scientific evidence does not allow for considering cumulative effects. The applicability of the acute reference dose (ARfD) of 1 μg ∆ ^9^-THC per kg body weight was re-confirmed by EFSA in 2020 ^30^ and by the German Federal Institute for Risk Assessment (BfR) in 2021 ^31^. The BfR has also concluded that the previously suggested German guidance values, which had been considered in versions 1–3 of this article, no longer correspond to current scientific knowledge ^31^. For this reason, the guidance values were removed from our assessment, which is now exclusively based on EFSA’s suggestions. For further details on interpretation of results and toxicity assessment, see Lachenmeier *et al.* ^2^. A detailed rationale for the estimation of the daily dose of products to be applied for the risk assessment has been provided in a correspondence article ^24^.

## Results and discussion

### Direct pharmacological effect of CBD as explanation of adverse effects

There is not much evidence to assume that chemically pure CBD may exhibit ∆ ^9^-THC-like adverse effects. The World Health Organization (WHO) judged the compound as being well tolerated with a good safety profile ^3^. Similar conclusions were made in a recent systematic review of CBD human trials ^32^.

CBD doses in the food supplements on the market are typically much lower than the ones tested in clinical studies. Additionally, there is a 90-day experiment in rats with a hemp extract (consisting of 26% cannabinoids, out of which 96% were CBD and less than 1% ∆ ^9^-THC) from which a NOAEL of 100 mg/kg bw/day could be derived ^33^. Based on 100 mg/kg bw/day × 26% × 96%, this would be about 25 mg/kg bw/day for CBD (or 1750 mg/day for a person with a body weight of 70 kg). This NOAEL would not typically be reached by the CBD dosages in food supplements.

On the other hand, there are still many uncertainties and contradictions remaining regarding cannabinoid safety studies ^34^. The metabolism of CBD is very complex. The main human metabolite is 7-carboxy-cannabidiol (7-COOH-CBD; ~90 % of all drug-related substances measured in the plasma), which may form a reactive acyl-glucuronide ^35– 37^. The toxicological profile of the metabolites has not been systematically investigated ^34^.

### CBD conversion into THC as explanation of adverse effects

Some, partly older, *in vitro* studies put up hypotheses about the conversion of CBD to ∆ ^9^-THC under acidic conditions such as in artificial gastric juice ^21, 38– 40^. If these proposals could be confirmed with *in vivo* data, consumers taking CBD orally could be exposed to such high ∆ ^9^-THC levels that the threshold for pharmacological action could be exceeded ^41^. However, taking a closer look at these *in vitro* studies raises some doubts. If CBD was to be converted to ∆ ^9^-THC *in vivo*, typical ∆ ^9^-THC metabolites should be detectable in blood and urine, but this has not been observed in oral or inhalatory CBD studies ^42– 44^. Due to the contradicting results, a replication of the *in vitro* study of Merrick *et al*. ^21^ was conducted using an extended experimental design. A more selective LC-MS/MS method and also an ultra-high pressure liquid chromatographic method with quadrupole time-of-flight mass spectrometry (UPLC-QTOF) were used to investigate the CBD degradation.

Under these conditions in contrast to Merrick *et al*. ^21^, no conversion of CBD to ∆ ^9^-THC was observed in any of the samples. Only in case of the positive control (2 week storage in 0.5 mol/L HCl and 50% methanol), a complete degradation of CBD into 27% ∆ ^9^-THC and other not identified products (with fragments similar to the ones found in cannabinol and ∆ ^9^-THC fragmentations but with other retention times) was observed (Table 1, underlying data ^23^). From an analytical viewpoint, the use of less selective and specific analytical methods, especially from the point of chromatographic separation, could result in a situation in which certain CBD degradation products might easily be confused with ∆ ^9^-THC due to structural similarities. Thus, similar fragmentation patterns and potentially overlapping peaks under certain chromatographic conditions might have led to false positive results in the previous studies. In conclusion of our degradation experiments, we agree with more recent literature ^45– 47^ that CBD would not likely react to ∆ ^9^-THC under *in vivo* conditions. The only detectable influence leading to degradation is strong acidity, which should be avoided in CBD formulations to ensure stability of products ^48^. Similar observations were recently provided by Yangsud et al. determining CBD as stabile under stress conditions, other than acidic or alkaline conditions ^49^. Transformation of CBD may also occur in acidified plasma samples or during pyrolysis gas chromatography ^50, 51^.

### ∆ ^9^-THC contamination as cause of adverse effects

Out of 181 samples, 21 samples (12% of the collective) have the potential to exceed the ∆ ^9^-THC LOAEL and were assessed as harmful to health. 82 samples (45% of the collective) were classified as unsuitable for human consumption due to exceeding the ARfD (see Table 2, underlying data ^23^). Furthermore, all food samples (i.e., all samples except CBD liquids intended to refill electronic cigarettes) have been classified as non-compliant to Regulation (EU) 2015/2283 of the European Parliament and of the Council of 25 November 2015 on novel foods ^52^ and therefore being unauthorized novel foods ^53^. The labelling of all food samples was also non-compliant to Regulation (EU) No 1169/2011 of the European Parliament and of the Council of 25 October 2011 on the provision of food information to consumers ^54^, e.g. due to lack of mandatory food information such as ingredients list or use of unapproved health claims in accordance to Regulation (EC) No 1924/2006 of the European Parliament and of the Council of 20 December 2006 on nutrition and health claims made on foods ^55^. In summary, none of the food products in our survey was found as being fully compliant with European food regulations.

The ∆ ^9^-THC dose leading to intoxication is considered to be in the range of 10 to 20 mg (very high dose in heavy episodic cannabis users up to 60 mg) for cannabis smoking ^56^. The resorption of orally ingested ∆ ^9^-THC varies greatly inter-individually with respect to both total amount and resorption rate ^57^. This might be one of the reasons for the individually very different observed psychotropic effects. A single oral dose of 20 mg THC resulted in symptoms such as tachycardia, conjunctival irritation, “high sensation” or dysphoria in adults within one to four hours. In one out of five adults, a single dose of 5 mg already showed corresponding symptoms ^58^.

Some of the CBD oil supplements contained ∆ ^9^-THC in doses up to 30 mg (in this case in the whole bottle of 10 ml), which can easily explain the adverse effects observed by some consumers. Interestingly, it was observed that the symptoms reported with cannabidiol exposures in the so far largest epidemiological study ^20^ were ∆ ^9^-THC-like symptoms ^59^.

Most of the CBD oils with dosage of around 1 mg ∆ ^9^-THC per serving offer the possibility to achieve intoxicating and psychotropic effects due to this compound if the products are used off-label (i.e. increase of the labelled maximum recommended daily dose by factors of 3–5, which is probably not an unlikely scenario. Some manufacturers even suggest an increase of daily dosage over time). Generally, these products pose a risk to human health considering EFSA’s ARfD that is considerably exceeded, even without consideration of THCA.

Hence our results provide compelling evidence that THC natively contained in CBD products may be a direct cause for adverse effects of these products. Obviously, there seems to be an involuntary or deliberate lack of quality control of CBD products. Claims of “THC-free”, used by most manufacturers, even on highly contaminated products – sometimes based on the use of unsuitable analytical methodologies with limits of detection in the percentage range –, have to be treated as fraudulent or deceptive food information.

## Conclusions

In light of the discussion about the three potential causative factors for adverse effects of CBD products, the described effects can be explained most probably by the presence of native THC as contaminant in the products rather than by direct action of CBD or its chemical transformation. The conclusions and findings of this study are further supported by the findings of Hazekamp ^6^ reporting data from the Netherlands on cannabis oils according to which the labelling information for CBD and ∆ ^9^-THC was often different from the actual contents. In 26 out of 46 products the ∆ ^9^-THC content was >1 g/100 mL. Further corresponding results were reported in a study from the USA, in which the CBD content was correctly declared for only 26 of 84 CBD products and 18 of the products had ∆ ^9^-THC contents >0.317 g/100 g ^60^.

CBD degradation products are currently unknown and need to be characterized and toxicologically assessed, e.g. within the context of the novel food authorisation process. Until then, the safety of the products remains questionable. Furthermore, standardization and purification of the extracts need to be improved and stability of commercial products during shelf life should be checked (e.g. to prevent CBD degradation by avoiding acidity in ingredients etc.).

In our opinion the systematically high ∆ ^9^-THC content of CBD products is clearly a “scandal” on the food market. Obviously, the manufacturers have – deliberately or in complete ignorance of the legal situation – placed unsafe and unapproved products on the market and thus exposed the consumer to an actually avoidable health risk. In view of the growing market for such lifestyle food supplements, the effectiveness of the instrument of food business operators’ own responsibility for food safety must obviously be challenged.

It has been claimed by C. Hillard that “many CBD products would be delivering enough THC along with it to provide a bit of a high and that’s more likely where the relief is coming from” ^61^. Our results have partially corroborated this opinion for a substantial number of products on the German market. Similarly, a recent survey reported that 22% out of 135 users of CBD products reported “feeling high” as common adverse effect ^12^. Nevertheless, the situation appears to have at least improved over time regarding extreme contents of THC: in 2020 only 4 samples were assessed as harmful to health, while in 2019 and 2018 this number was 10 and 7, respectively. A statistically significant decline of THC levels over all CBD products over the three years was not provable, however.

Currently we still observe a CBD market in the EU, where obviously considerable numbers of unsafe and misleadingly labelled products are available. Due to consistent deficits in mandatory labelling including a lack of maximum recommended daily dose, dosages up to psychotropic levels (for THC) or pharmacological levels (for CBD) cannot be excluded with certainty. The risk also includes positive cannabis urine tests for several days, which may be expected from daily oral doses of more than 1 mg ∆ ^9^-THC ^1, 2, 62^. Therefore, about 15% of products in our study would probably lead to false-positive urine tests, which could have grave consequences for persons occupationally or otherwise required to prove absence of drug use or of doping in professional sports ^63– 65^.

Obviously, the current regulatory framework is insufficient to adequately regulate products in the grey area between medicines and food supplements. For cannabis-derived products, such as CBD, the problem is aggravated by conflicting regulations in the narcotic, medicinal, and food law areas. For example, hemp extract-based products of similar composition were suggested to be treated as illegal narcotics, prescription-based medicinal products, or novel foods. Only recently, the EU commission clarified its position to not further consider cannabidiol as narcotic, but to advance the novel food approval procedure ^66^. Clearly for CBD products alongside other cannabis products, a regulated legalization (see e.g. Anderson *et al.* ^67^) would be preferable, introducing stricter regulations, such as mandatory labelling requirements, safety assessment, testing, pre-marketing approval and post-marketing surveillance (also see 43, 68).

## Data availability

### Underlying data

Open Science Framework: Dataset for “Are adverse effects of cannabidiol (CBD) products caused by delta9-tetrahydrocannabinol (THC) contamination?” (Version 3) https://doi.org/10.17605/OSF.IO/F7ZXY ^23^

This project contains the following underlying data:

- Dataset for 'Are adverse effects of cannabidiol (CBD) products caused by delta9-tetrahydrocannabinol (THC) contamination' F1000 Research.xlsx (Version 3) (Excel spreadsheet with data underlying Table 1 and Table 2, missing data/empty cells correspond to values outside calibration (CBD) or not measured)

Data are available under the terms of the Creative Commons Zero “No rights reserved” data waiver (CC0 1.0 Public domain dedication).
