# Are adverse effects of cannabidiol (CBD) products caused by tetrahydrocannabinol (THC) contamination?

**DOI:** 10.12688/f1000research.19931.5

**Published:** 2022-11-10

**Authors:** Dirk W. Lachenmeier, Stephanie Habel, Berit Fischer, Frauke Herbi, Yvonne Zerbe, Verena Bock, Tabata Rajcic de Rezende, Stephan G. Walch, Constanze Sproll

**Affiliations:** 1 Chemisches und Veterinäruntersuchungsamt (CVUA) Karlsruhe, Karlsruhe, 76187, Germany

**Keywords:** Tetrahydrocannabinol, cannabidiol, Cannabis sativa, hemp, food supplements, risk assessment, drug effects

## Abstract

Cannabidiol (CBD)-containing products are widely marketed as over the counter products, mostly as food supplements. Adverse effects reported in anecdotal consumer reports or during clinical studies were first assumed to be due to hydrolytic conversion of CBD to psychotropic Δ ^9^-tetrahydrocannabinol (Δ ^9^-THC) in the stomach after oral consumption. However, research of pure CBD solutions stored in simulated gastric juice or subjected to various storage conditions such as heat and light with specific liquid chromatographic/tandem mass spectrometric (LC/MS/MS) and ultra-high pressure liquid chromatographic/quadrupole time-of-flight mass spectrometric (UPLC-QTOF) analyses was unable to confirm THC formation. Another hypothesis for the adverse effects of CBD products may be residual Δ ^9^-THC concentrations in the products as contamination, because most of them are based on hemp extracts containing the full spectrum of cannabinoids besides CBD. Analyses of 293 food products of the German market (mostly CBD oils) confirmed this hypothesis: 28 products (10%) contained Δ ^9^-THC above the lowest observed adverse effect level (2.5 mg/day). Hence, it may be assumed that the adverse effects of some commercial CBD products are based on a low-dose effect of Δ ^9^-THC, with the safety of CBD itself currently being unclear with significant uncertainties regarding possible liver and reproductive toxicity. The safety, efficacy and purity of commercial CBD products is highly questionable, and all of the products in our sample collection showed various non-conformities to European food law such as unsafe Δ ^9^-THC levels, hemp extracts or CBD isolates as non-approved novel food ingredients, non-approved health claims, and deficits in mandatory food labelling requirements. In view of the growing market for such lifestyle products, the effectiveness of the instrument of food business operators' own responsibility for product safety and regulatory compliance must obviously be challenged, and a strong regulatory framework for hemp products needs to be devised.

## Introduction

Since hemp has been re-approved for cultivation as an industrial crop in the form of low Δ ^9^-tetrahydrocannabinol (∆ ^9^-THC) hemp ( *Cannabis sativa* L.) varieties in the European Union (EU), components of the hemp plant are increasingly used for the production of foods and other consumer products such as liquids for electronic cigarettes ^1^.

From all hemp constituents, cannabidiol (CBD) is currently the compound with highest interest. In contrast to ∆ ^9^-THC, the major narcotic constituent of hemp, CBD is a non-psychotropic cannabinoid. It is currently being tested for its possible antispasmodic, anti-inflammatory, anxiolytic and antiemetic effects as a drug, e.g. for the treatment of epilepsy ^2, 3^. However, CBD products of all kinds can now also be purchased in organic shops, drug stores, specialised hemp and CBD stores, but also in supermarkets and via the Internet, often by advertising questionable “cure-all” properties including various unspecific health advantages. The marketing of CBD products is based on the current “hype” around medicinal hemp products, whereby the CBD products are offered as a supposedly safe alternative, promised as being free of psychotropic components or their adverse effects ^4^. The awareness of recreational CBD use in Germany is high, with approximately half of the population being aware of them and 4.3% of the population having ever used them (1.1% current users) ^5^. With the exception of the treatment of Dravet’s syndrome, there is little clinical data on the efficacy and safety of CBD ^6, 7^. Cannabidiol is currently approved in the EU in a single medicinal product, namely Epidiolex® for the treatment of seizures in patients with two rare, severe forms of childhood-onset epilepsy. Apart from that, extemporaneous preparations in pharmacies are legally available on prescription in Germany and some other countries. However, most of the CBD products worldwide are available as food supplements or CBD-containing hemp extracts are used as ingredient in other foods.

Commercial CBD products are usually crude extracts from whole hemp plants (i.e., including flowers and stems). In other ways (e.g., in extracting the food-approved plant parts such as seeds), contents in the range of 1–10% CBD, which are typically advertised, cannot be achieved. Also, the limited available literature and manufacturer data confirm that CBD products are usually extracted by supercritical CO _2_ or with solvents such as ethanol or isopropanol from the entire hemp plant material, which typically has been decarboxylated before the process ^7, 8^. No further specific enrichment or purification of CBD is often conducted, so that the commercial extracts are regularly a cannabinoid mixture rather than pure CBD. Otherwise, extracts may be cleaned with different processes such as winterization, or partial fractionation using supercritical CO _2_. These extracts, which are typically called “full spectrum extracts” in difference to chemically pure CBD (such as isolated or synthesized CBD), are then mixed into ordinary edible oils such as sunflower oil, olive oil or hemp seed oil to obtain the so-called CBD oil ^7^.

The strategy to market CBD products as food supplements within the framework of food regulations seems to be the most common approach of CBD sellers. The most prevalent food supplement products are CBD oils in liquid form or hemp extract containing capsules. Some other products, derived from hemp extracts, are CBD chewing gum, and cannabis resin, wax or pollen products, while so-called “CBD flowers” are typically sold as plant material to prepare a tea-like infusion or as an herbal product for smoking.

However, no significant food consumption of CBD products has been documented before 15 May 1997. These products are therefore classified as “novel” in the Novel Food catalogue of the European Commission under the entry “cannabinoids” and therefore require approval according to the Novel Food Regulation. Up to date (as of October 2022), no approved application is documented. Basically, all available CBD products based on hemp extract but also those based on isolated or synthesized CBD, which are marketed as food or food supplement within the EU, are therefore illegally sold ^2^. To circumvent the strict safety requirements for medicinal or food products, some CBD products may be sold as other product categories (e.g., cosmetics, veterinary supplements, waxes, flavourings, air fresheners or room fragrances), but the off-label use, human consumption, is clearly intended.

Despite the enforcement efforts of the food and medicinal product control authorities (e.g. the EU’s rapid alert system for food and feed (RASFF) lists over 180 alerts for CBD since 2018), a multitude of CBD products is available on the internet and in some retail stores, so that CBD is currently easily available to consumers.

Despite the lack of mandatory nutrivigilance in the EU, anecdotal cases ranging from indisposition to ∆ ^9^-THC-like effects have been reported to our institute from food control authorities in the German Federal State of Baden-Württemberg in the context of consumer complaint cases regarding CBD products. Several case reports of adverse effects of CBD products have also been published ^9– 12^, and a survey of 135 CBD users in the USA detected a high prevalence of adverse effects (30% dry mouth, 22% feeling high, 20% change in appetite, 19% fatigue) ^13^. Additionally, some paediatric studies in epilepsy patients with orally administered CBD also reported adverse effects such as drowsiness and fatigue that could be explained by pharmacological properties of ∆ ^9^-THC rather than of CBD ^14– 16^. Respiratory depression was reported in a case of CBD overdose in a paediatric patient ^17^. Clinical trials with doses of 300 mg/day and above have shown elevated liver enzymes consistent with liver injury ^18, 19^. Concerns also include negative effects on the male reproductive system ^20^.

Diarrhoea was an adverse outcome associated with CBD treatment in a meta-analysis of randomized clinical trials, after excluding studies of childhood epilepsy ^21^. Post marketing safety surveillance of a full spectrum hemp extract reported gastrointestinal symptoms as most common adverse effect, however, they were infrequent (0.03%) ^22^. More recently, the epidemiology of CBD-related cases in the national poison data system of the USA was assessed. Cannabidiol cases increased from 0% in 2009–2018 to 17% of all cases in 2019 ^23^. Among the exposures in which CBD was the only reported substance (n=1275), the most common symptoms were mild central nervous system depression (10%), tachycardia (6%), dizziness/vertigo (5%), vomiting (5%), nausea (5%) and agitation (4%) ^24^. The European food safety authority (EFSA) has recently summarised the state of knowledge on the safety of CBD consumption in the context of the novel food approval procedures. The EFSA determined that the effect of CBD on liver, gastrointestinal tract, endocrine system, nervous system and on psychological function needs to be clarified, and that studies in animals show significant reproductive toxicity ^25^.

Currently there are three hypotheses for the cause of the adverse effects: (i) a direct pharmacological effect of CBD, (ii) the degradation of CBD to ∆ ^9^-THC due to acidic hydrolysis in the stomach following oral consumption, and (iii) ∆ ^9^-THC directly contained in the products as by-product due to co-extraction and enrichment or contamination such as formation from CBD degradation during storage. In this article, the hypotheses are investigated including new evidence from original data.

## Methods

### CBD degradation

To investigate CBD degradation into ∆ ^9^-THC under acidic conditions, differently concentrated CBD in methanolic solutions was used in a range corresponding to typical amounts consumed with supplements based on commercial CBD (Supelco Cerilliant, certified reference material, #C-045, 1.0 mg/mL in methanol) supplied by Merck (Darmstadt, Germany). These solutions were exposed to an artificial gastric juice as well as different incubation times and stress factors such as storage under light and heat (see Table 1 for full experimental design). The solutions were stored either in standard freezer (-18°C) or refrigerator (8°C) or at room temperature (20°C). Increased temperatures were achieved using a thermostatically controlled laboratory drying oven type “UT6120” (Heraeus, Langenselbold, Germany) set to either 37°C or 60°C. The daylight condition was achieved by storage at a window (south side). For ultraviolet light exposure, six 25 W ultraviolet (UV) fluorescent tubes type “excellent E” (99.1% UVA) built into a facial tanner type “NT 446 U” (Dr. Kern GmbH, Mademühlen, Germany) were placed 15 cm from the surface of the solutions (open sample vials). In deviation of an experimental protocol of Merrick *et al*. ^26^, a gastric juice without addition of surfactants was used, which was strictly produced according to the European pharmacopoeia ^27^ (0.020 g NaCl + 0.032 g pepsin + 0.8 mL HCl (1 mol/L), filled up to 10 mL with water). As pure CBD was available only in methanolic solution, the final experimental setups contained 0.08 mol/L HCl and 1% methanol due to dilution (methanol residues in this order of magnitude are not interfering with the analysis).

**Table 1.** Cannabidiol (CBD) stability experiments under various storage conditions.

Experiment	Temperature (°C)	Light exposure	Storage time	Storage medium	CBD concentration in medium (μg/L)	Δ ^9^-THC formation ^1^
Negative control	-18	None	14 days	Methanol	1000	0%
Light	20	None	3 days	Methanol	1000	0%
	20	None	14 days	Methanol	1000	0%
	20	Daylight	3 days	Methanol	1000	0%
	20	Daylight	14 days	Methanol	1000	0%
	20	UVA	1 h	Methanol	1000	0%
	20	UVA	3 h	Methanol	1000	0%
Temperature	20	None	5 days	Methanol	1000	0%
	20	None	14 days	Methanol	1000	0%
	8	None	5 days	Methanol	1000	0%
	8	None	14 days	Methanol	1000	0%
	37	None	3 h	Methanol	1000	0%
	60	None	1 h	Methanol	1000	0%
Simulated gastric juice	37	None	1 h	Simulated gastric juice	200	0%
	37	None	2 h	Simulated gastric juice	200	0%
	37	None	3 h	Simulated gastric juice	200	0%
	37	None	1 h	Simulated gastric juice	400	0%
	37	None	2 h	Simulated gastric juice	400	0%
	37	None	3 h	Simulated gastric juice	400	0%
Positive control	20	None	14 days	Methanol / 1 mol/L HCl (50:50)	500	27%

^1^ Average of LC-MS/MS and UPLC-QTOF measurements (n=2) (for raw results see dataset ^28^, table sheet 1). Δ ^9^-THC formation calculated as % in relation to original CBD content.

Abbreviations: CBD: cannabidiol; Δ ^9^-THC: Δ ^9^-tetrahydrocannabinol; UVA: ultraviolet A; LC-MS/MS: liquid chromatography/tandem mass spectrometry; UPLC-QTOF: ultra-high pressure liquid chromatography/quadrupole time-of-flight mass spectrometry

To ensure the utmost analytical validity, all samples were independently measured on two different instruments, using a triple quadrupole mass spectrometer (TSQ Vantage, Thermo Fisher Scientific, San Jose, CA, USA) coupled with an LC system (1100 series, Agilent, Waldbronn, Germany) and also using a quadrupole time-of-flight (QTOF) mass spectrometer (X500, Sciex, Darmstadt, Germany) coupled with an UPLC system (1290 series, Agilent, Waldbronn, Germany). Both systems used the same type of separation column (Luna Omega Polar C18, 150 × 2.1 mm, 1.6 μm, 100 Å, Phenomenex, Aschaffenburg, Germany). The separation was isocratic with 25 % water (0.1 % formic acid) and 75 % acetonitrile (0.1 % formic acid) and a flow of 0.3 mL/min. In case of QTOF with 35 % water (0.1 % formic acid) and 65 % acetonitrile (0.1 % formic acid) and a flow of 0.45 mL/min. The evaluation took place after fragmentation of the mother ion into three mass traces for each compound. As quantifier for ∆ ^9^-THC and CBD, the mass transition m/z 315 to 193 was used. In case of QTOF, quantification was conducted over accurate mass and control of fragmentation pattern. CBD eluted as one of the first cannabinoids, a few minutes before ∆ ^9^-THC. As internal standards ∆ ^9^-THC-d _3_ (Supelco Cerilliant #T-011, 1.0 mg/mL in methanol) was used for the quantification of ∆ ^9^-THC (Supelco Cerilliant #T-005, 1.0 mg/mL in methanol), and cannabidiol-d _3_ (Supelco Cerilliant #C-084, 100 μg/mL in methanol) for quantification of CBD (Supelco Cerilliant #C-045, 1.0 mg/mL in methanol). The certified reference materials were obtained as solutions in ampoules of 1 mL, all supplied by Merck (Darmstadt, Germany). A limit of detection (LOD) of 5 ng/mL was determined. For both procedures, relative standard deviations better than 5% were achieved. Both methods are able to chromatographically separate ∆ ^9^-THC and CBD from their acids. Specificity was ensured using a certified reference material as a reference standard of THCA (Supelco Cerilliant #T-093, 1.0 mg/mL in acetonitrile). Baseline separation was achieved between ∆ ^9^-THC, ∆ ^8^-THC and THCA. Therefore, the reported values in this study are specific for ∆ ^9^-THC and CBD. In contrast to some previous studies based on gas chromatography, we do not report “total THC” or “total CBD”, which would be a sum of the free form and its acid.

### ∆ ^9^-THC contamination of commercial products

To study the possible influence of natively contained ∆ ^9^-THC in hemp products as a cause for adverse effects, a sampling of available CBD products registered as food supplement in the German State Baden-Württemberg, other available hemp extract products in retail, as well as all products available at the warehouse of a large internet retailer were sampled between December 2018 and December 2021. A total of 293 samples (see Table 2) were analysed using the above-described liquid chromatographic method with tandem mass spectrometric detection (LC-MS/MS) for ∆ ^9^-THC content. For 2020-2021 samples, the following parameters of the method were changed: separation column (Raptor, ARC-18, 150 × 2.1 mm, 2.7 μm, Restek, Bad Homburg, Germany). The separation was a gradient starting with 20% eluent A (0.1 % formic acid in water) and 80% eluent B (0.1 % formic acid in methanol) for 18 min, followed by 5% A and 95% B for 5 min, and back to 20% A and 80% B for 7 min. All methods were validated and externally accredited according to ISO 17025 standard. Recently, the method reported satisfactory results for ∆ ^9^-THC during the international government chemist CBD food and cosmetic ring trial ^29^.

**Table 2.** Results ^1^ of THC analysis in commercial hemp-based products from the German market (2018–2021).

Year	Samples with Δ ^9^-THC content exceeding LOAEL	Samples with Δ ^9^-THC content between ARfD and LOAEL	Sample with Δ ^9^-THC content below ARfD	Samples (total)
2018	7 (78%)	2 (22%)	0 (0%)	9
2019	10 (16%)	30 (47%)	24 (38%)	64
2020	4 (4%)	49 (46%)	54 (50%)	107
2021	7 (6%)	50 (44%)	56 (50%)	113
2018–2021 (total)	28 (10%)	131 (45%)	134 (46%)	293

^1^ For raw results see dataset ^28^, table sheet 2. Abbreviations: Δ ^9^-THC: Δ ^9^-tetrahydrocannabinol; ARfD: acute reference dose of 1 μg THC per kg body weight ^30^; LOAEL: lowest observed adverse effect level of 2.5 mg Δ ^9^-THC per day ^30^

For toxicological evaluation of the results, the lowest observed adverse effect level (LOAEL) of 2.5 mg ∆ ^9^-THC per day published by the EFSA based on human data (central nervous system effects and pulse increase) was used ^30^. Taking uncertainty factors (factor 3 for extrapolation from LOAEL to no observed adverse effect level (NOAEL) and factor 10 for interindividual differences, total factor 30) into account, an acute reference dose (ARfD) of 1 μg ∆ ^9^-THC per kg body weight was derived ^30^. In their assessment, the Panel on Contaminants in the Food Chain of EFSA also considered interaction between ∆ ^9^-THC and CBD, but found the information controversial and not consistently antagonistic ^30^. This is consistent with more recent research of Solowij *et al.* ^31^ that the effects of ∆ ^9^-THC may even be enhanced by low-dose CBD (e.g., as found in food supplements) and may be particular prominent in infrequent cannabis users. However, the current scientific evidence does not allow for considering cumulative effects. The applicability of the acute reference dose (ARfD) of 1 μg ∆ ^9^-THC per kg body weight was re-confirmed by EFSA in 2020 ^32^ and by the German Federal Institute for Risk Assessment (BfR) in 2021 ^33^. The BfR has also concluded that the previously suggested German guidance values, which had been considered in versions 1–3 of this article, no longer correspond to current scientific knowledge ^33^. For this reason, the guidance values were removed from our assessment, which is now exclusively based on EFSA’s suggestions. For further details on interpretation of results and toxicity assessment, see Lachenmeier *et al.* ^2^. A detailed rationale for the estimation of the daily dose of products to be applied for the risk assessment has been provided in a correspondence article ^34^.

## Results and discussion

### Direct pharmacological effect of CBD as explanation of adverse effects

There is not much evidence to assume that chemically pure CBD may exhibit acute ∆ ^9^-THC-like adverse effects. The World Health Organization (WHO) judged the compound as being well tolerated with a good safety profile ^3^. Similar conclusions were made in a recent systematic review of CBD human trials ^35^.

CBD doses in the food supplements on the market are typically much lower than the ones tested in clinical studies. Nevertheless, the EFSA judged in their review of available human and animal studies that a NOAEL could not be identified ^25^, and that there might be a possible risk of long-term effects in humans from chronic consumption of CBD as food. To exclude such chronic effect, based on the LOAEL for CBD of 4.3 mg/kg bw/day (or 300 mg/day for a person with a body weight of 70 kg) for liver effects in humans ^25, 36^, a health-based guidance value (HBGV) of 10 mg/day might be assumed using the uncertainty factor of 30 similar to the evaluation of THC ^36^. This HBGV could be exceeded by the CBD dosages in some of the food supplements.

Additionally, there are still many uncertainties and contradictions remaining regarding cannabinoid safety studies ^37^. The metabolism of CBD is very complex. The main human metabolite is 7-carboxy-cannabidiol (7-COOH-CBD; ~90 % of all drug-related substances measured in the plasma), which may form a reactive acyl-glucuronide ^38– 40^. Similar to CBD itself, the toxicological profile of its metabolites has not been systematically investigated ^37^.

### CBD conversion into THC as explanation of adverse effects

Some, partly older, *in vitro* studies put up hypotheses about the conversion of CBD to ∆ ^9^-THC under acidic conditions such as in artificial gastric juice ^26, 41– 43^. If these proposals could be confirmed with *in vivo* data, consumers taking CBD orally could be exposed to such high ∆ ^9^-THC levels that the threshold for pharmacological action could be exceeded ^44^. However, taking a closer look at these *in vitro* studies raises some doubts. If CBD was to be converted to ∆ ^9^-THC *in vivo*, typical ∆ ^9^-THC metabolites should be detectable in blood and urine, but this has not been observed in oral or inhalatory CBD studies ^45– 47^. Due to the contradicting results, a replication of the *in vitro* study of Merrick *et al*. ^26^ was conducted using an extended experimental design. A more selective LC-MS/MS method and also an ultra-high pressure liquid chromatographic method with quadrupole time-of-flight mass spectrometry (UPLC-QTOF) were used to investigate the CBD degradation.

Under these conditions in contrast to Merrick *et al*. ^26^, no conversion of CBD to ∆ ^9^-THC was observed in any of the samples. Only in case of the positive control (2 week storage in 0.5 mol/L HCl and 50% methanol), a complete degradation of CBD into 27% ∆ ^9^-THC and other not identified products (with fragments similar to the ones found in cannabinol and ∆ ^9^-THC fragmentations but with other retention times) was observed (Table 1, underlying data ^28^). From an analytical viewpoint, the use of less selective and specific analytical methods, especially from the point of chromatographic separation, could result in a situation in which certain CBD degradation products might easily be confused with ∆ ^9^-THC due to structural similarities. Thus, similar fragmentation patterns and potentially overlapping peaks under certain chromatographic conditions might have led to false positive results in the previous studies. In conclusion of our degradation experiments, we agree with more recent literature ^48– 51^ that CBD would not likely react to ∆ ^9^-THC under *in vivo* conditions. The only detectable influence leading to degradation is strong acidity, which should be avoided in CBD formulations to ensure stability of products ^52^. Similar observations were recently provided by Yangsud *et al.* determining CBD as stabile under stress conditions, other than acidic or alkaline conditions ^53^. Transformation of CBD may also occur in acidified plasma samples or during pyrolysis gas chromatography ^54, 55^, but not during vaping or smoking of low-THC cannabis products ^56^.

### ∆ ^9^-THC contamination as cause of adverse effects

Out of 293 samples, 28 samples (10% of the collective) have the potential to exceed the ∆ ^9^-THC LOAEL and were assessed as harmful to health. 131 samples (45% of the collective) were classified as unsuitable for human consumption due to exceeding the ARfD (see Table 2, underlying data ^28^). Furthermore, all food samples (i.e., all samples except CBD liquids intended to refill electronic cigarettes) have been classified as non-compliant to Regulation (EU) 2015/2283 of the European Parliament and of the Council of 25 November 2015 on novel foods ^57^ and therefore being unauthorized novel foods ^58^. The labelling of all food samples was also non-compliant to Regulation (EU) No 1169/2011 of the European Parliament and of the Council of 25 October 2011 on the provision of food information to consumers ^59^, e.g. due to lack of mandatory food information such as ingredients list or use of unapproved health claims in accordance to Regulation (EC) No 1924/2006 of the European Parliament and of the Council of 20 December 2006 on nutrition and health claims made on foods ^60^. In summary, none of the food products in our survey was found as being fully compliant with European food regulations.

The ∆ ^9^-THC dose leading to intoxication is considered to be in the range of 10 to 20 mg (very high dose in heavy episodic cannabis users up to 60 mg) for cannabis smoking ^61^. The resorption of orally ingested ∆ ^9^-THC varies greatly inter-individually with respect to both total amount and resorption rate ^62^. This might be one of the reasons for the individually very different observed psychotropic effects. A single oral dose of 20 mg THC resulted in symptoms such as tachycardia, conjunctival irritation, “high sensation” or dysphoria in adults within one to four hours. In one out of five adults, a single dose of 5 mg already showed corresponding symptoms ^63^.

Some of the CBD oil supplements contained ∆ ^9^-THC in doses up to 30 mg (in this case in the whole bottle of 10 ml), which can easily explain the adverse effects observed by some consumers. Interestingly, it was observed that the symptoms reported with cannabidiol exposures in the so far largest epidemiological study ^24^ were ∆ ^9^-THC-like symptoms ^64^.

Most of the CBD oils with dosage of around 1 mg ∆ ^9^-THC per serving offer the possibility to achieve intoxicating and psychotropic effects due to this compound if the products are used off-label (i.e. increase of the labelled maximum recommended daily dose by factors of 3–5, which is probably not an unlikely scenario. Some manufacturers even suggest an increase of daily dosage over time). Generally, these products pose a risk to human health considering EFSA’s ARfD that is considerably exceeded, even without consideration of THCA.

Hence our results provide compelling evidence that THC natively contained in CBD products may be a direct cause for adverse effects of these products. Obviously, there seems to be an involuntary or deliberate lack of quality control of CBD products. Claims of “THC-free”, used by most manufacturers, even on highly contaminated products – sometimes based on the use of unsuitable analytical methodologies with limits of detection in the percentage range –, have to be treated as fraudulent or deceptive food information.

## Conclusions

In light of the discussion about the three potential causative factors for adverse effects of CBD products, the described effects can be explained most probably by the presence of native THC as contaminant in the products rather than by direct action of CBD or its chemical transformation. The conclusions and findings of this study are further supported by several other surveys from the Netherlands and the USA showing inconsistent labelling and THC contents ^7, 65– 67^.

CBD degradation products are currently unknown and need to be characterized and toxicologically assessed, e.g. within the context of the novel food authorisation process. Until then, the safety of the products remains questionable. Furthermore, standardization and purification of the extracts need to be improved and stability of commercial products during shelf life should be checked (e.g. to prevent CBD degradation by avoiding acidity in ingredients etc.).

In our opinion the systematically high ∆ ^9^-THC content of CBD products is clearly a “scandal” on the food market. Obviously, the manufacturers have – deliberately or in complete ignorance of the legal situation – placed unsafe and unapproved products on the market and thus exposed the consumer to an actually avoidable health risk. In view of the growing market for such lifestyle food supplements, the effectiveness of the instrument of food business operators’ own responsibility for food safety must obviously be challenged.

It has been claimed by C. Hillard that “many CBD products would be delivering enough THC along with it to provide a bit of a high and that’s more likely where the relief is coming from” ^68^. Our results have partially corroborated this opinion for a substantial number of products on the German market. Similarly, a recent survey reported that 22% out of 135 users of CBD products reported “feeling high” as common adverse effect ^13^. 

Currently we still observe a CBD market in the EU, where obviously considerable numbers of unsafe and misleadingly labelled products are available. Due to consistent deficits in mandatory labelling including a lack of maximum recommended daily dose, dosages up to psychotropic levels (for THC) or pharmacological levels (for CBD) cannot be excluded with certainty. The risk also includes positive cannabis urine tests for several days, which may be expected from daily oral doses of more than 1 mg ∆ ^9^-THC ^1, 2, 69^. Therefore, about 16% of products in our study would probably lead to false-positive urine tests, which could have grave consequences for persons occupationally or otherwise required to prove absence of drug use or of doping in professional sports ^70– 72^. Possible long-term risks encompass liver toxicity and reproductive toxicity ^25^.

Obviously, the current regulatory framework is insufficient to adequately regulate products in the grey area between medicines and food supplements. For cannabis-derived products, such as CBD, the problem is aggravated by conflicting regulations in the narcotic, medicinal, and food law areas. For example, hemp extract-based products of similar composition were suggested to be treated as illegal narcotics, prescription-based medicinal products, or novel foods. Only recently, the EU commission clarified its position to not further consider cannabidiol as narcotic, but to advance the novel food approval procedure ^73^. Clearly for CBD products alongside other cannabis products, a regulated legalization (see e.g. Anderson *et al.* ^74^) would be preferable, introducing stricter regulations, such as mandatory labelling requirements, safety assessment, testing, pre-marketing approval and post-marketing surveillance (also see 46, 75).

## Data Availability

Open Science Framework: Dataset for “Are adverse effects of cannabidiol (CBD) products caused by delta9-tetrahydrocannabinol (THC) contamination?” (Version 4) https://doi.org/10.17605/OSF.IO/F7ZXY ^28^ This project contains the following underlying data: Dataset for 'Are adverse effects of cannabidiol (CBD) products caused by delta9-tetrahydrocannabinol (THC) contamination' F1000 Research.xlsx (Version 4) (Excel spreadsheet with data underlying Table 1 and Table 2, missing data/empty cells correspond to values outside calibration (CBD) or not measured) Data are available under the terms of the Creative Commons Zero “No rights reserved” data waiver (CC0 1.0 Public domain dedication).
