# Supplementary material for: Are adverse effects of cannabidiol (CBD) products caused by tetrahydrocannabinol (THC) contamination?
Source: F1000Res. 2024 Sep 13;8:1394. Originally published 2019 Aug 8. [Version 7] doi: 10.12688/f1000research.19931.7 (PMC7029751; doi:10.12688/f1000research.19931.7)
Supplement: Peer review history

# Reviewer report: Reviewer response for version 7

**DOI:** 10.5256/f1000research.170935.r323274

**Published:** 2024-09-14

**Authors:** Volker Auwärter

**Affiliations:** 1 Institute of Forensic Medicine, Forensic Toxicology, Medical Center - University of Freiburg, Freiburg, Germany

The revision is fine.

Is the work clearly and accurately presented and does it cite the current literature?

Partly

If applicable, is the statistical analysis and its interpretation appropriate?

Not applicable

Are all the source data underlying the results available to ensure full reproducibility?

Partly

Is the study design appropriate and is the work technically sound?

Partly

Are the conclusions drawn adequately supported by the results?

Partly

Are sufficient details of methods and analysis provided to allow replication by others?

Partly

Reviewer Expertise:

Forensic Toxicology

I confirm that I have read this submission and believe that I have an appropriate level of expertise to confirm that it is of an acceptable scientific standard.

# Reviewer report: Reviewer response for version 5

**DOI:** 10.5256/f1000research.140541.r155511

**Published:** 2022-11-11

**Authors:** Arno Hazekamp

**Affiliations:** 1 Hazekamp Herbal Consulting BV, Leiden, The Netherlands

Reviewer Expertise:

Analytical science, cannabis, cannabinoids, quality control

I confirm that I have read this submission and believe that I have an appropriate level of expertise to confirm that it is of an acceptable scientific standard.

# Reviewer report: Reviewer response for version 3

**DOI:** 10.5256/f1000research.28743.r69794

**Published:** 2020-08-24

**Authors:** Arno Hazekamp

**Affiliations:** 1 Hazekamp Herbal Consulting BV, Leiden, The Netherlands

Great job done by the authors. This manuscript is ready for indexing.

Reviewer Expertise:

medicinal cannabis cultivation, quality control, development of administration forms, clinical trials, patient surveys.

I confirm that I have read this submission and believe that I have an appropriate level of expertise to confirm that it is of an acceptable scientific standard.

# Reviewer report: Reviewer response for version 2

**DOI:** 10.5256/f1000research.24583.r59941

**Published:** 2020-07-06

**Authors:** Volker Auwärter

**Affiliations:** 1 Institute of Forensic Medicine, Forensic Toxicology, Medical Center - University of Freiburg, Freiburg, Germany

With respect to the two previous reviews I will refrain from summarizing the article once more.

 Although the article addresses an important issue and the hypothesis of THC residues being responsible for some of the adverse effects of CBD preparations is plausible and supported by the data, I miss the discussion of two relevant points:

1. No details are given regarding the extraction of the products. Was the 'total THC content' measured by heat-induced decarboxylation of THC acid A? That would be close to the procedure used by most forensic laboratories in Germany. The author's response to reviewer 2 (page 4, comments #3 and #8) suggests, however, that THC acid A was determined separately. If the 'total THC content' was given as the sum of THC and THC acid A after correction for the molecular weight the dose would be overestimated (maximum conversion rates of smoked, 'vaped' or baked cannabis were usually reported to be below 70%). The available THC doses after oral ingestion without heating the material would be even lower (depending on the THC acid A content).
2. Regarding the THC dose required to produce psychotropic effects the authors did not discuss the 'inverse agonist' like properties of CBD at the CB1 receptor (McPartland *et al.*, 2015 ^1^) which have been shown to reduce the intoxicating effects of THC (e.g. Solowij *et al.*, 2019 ^2^). This might affect the LOAEL and the ARfD of THC when contained in CBD rich products as these values were not yet assessed for such cannabinoid preparations.

Page 8: The German guidance value seems to be several orders of magnitude (up to 4) lower than the CBD contents in the products.

Is the work clearly and accurately presented and does it cite the current literature?

Partly

If applicable, is the statistical analysis and its interpretation appropriate?

Not applicable

Are all the source data underlying the results available to ensure full reproducibility?

Partly

Is the study design appropriate and is the work technically sound?

Partly

Are the conclusions drawn adequately supported by the results?

Partly

Are sufficient details of methods and analysis provided to allow replication by others?

Partly

Reviewer Expertise:

Forensic Toxicology, Metabolism, NPS, Cannabinoids

I confirm that I have read this submission and believe that I have an appropriate level of expertise to confirm that it is of an acceptable scientific standard, however I have significant reservations, as outlined above.

## References

[1] : Are cannabidiol and Δ(9) -tetrahydrocannabivarin negative modulators of the endocannabinoid system? A systematic review. *Br J Pharmacol* .2015;172(3) : 10.1111/bph.12944 737-53 10.1111/bph.12944 25257544 PMC4301686

[2] : A randomised controlled trial of vaporised Δ9-tetrahydrocannabinol and cannabidiol alone and in combination in frequent and infrequent cannabis users: acute intoxication effects. *Eur Arch Psychiatry Clin Neurosci* .2019;269(1) : 10.1007/s00406-019-00978-2 17-35 10.1007/s00406-019-00978-2 30661105

# Response

**Published:** 2020-07-09

**Authors:** Dirk W. Lachenmeier

**Affiliations:** Chemisches und Veterinäruntersuchungsamt (CVUA) Karlsruhe, Germany

Thank you for your comments!

1. Regarding the extraction and measurement of the products, no heat was applied during the whole procedure. The samples were extracted and/or diluted using solvents at room temperature and then subjected to LC-MS/MS as described in the methods section. By this method, tetrahydrocannabinolic acid (THCA) can be distinguished from both ∆ ^9^-THC and ∆ ^8^-THC (the methods section in version 1 of the paper is more detailed in this regard). As THCA is not psychotropic, we believe that the use of “total THC content”, which has been historically based on gas chromatographic determination always leading to decarboxylation in the injection port, is not informative for meaningful risk assessment of cannabis products (for details on risk assessment see ^1^). Hence, we only report the specific content of ∆ ^9^-THC in Table 2 of our article (in our article THC is used as abbreviation for Δ ^9^-tetrahydrocannabinol but not for “total THC”, see introduction line 2). The many samples that exceed the German guideline value (which – as footnote 6 in table 2 correctly states – indeed refers to total THC), exceed it already for ∆ ^9^-THC alone. Therefore, we have disregarded THCA for this assessment and refrained from calculating sums of THC and THCA. This means that the assumption of the reviewer that we might have overestimated the risk by including THCA is unfounded. Considering the German guideline values, our approach purely based on ∆ ^9^-THC is even for the benefit of the manufacturer, as we believe it is over-conservative to include precursors of  ∆ ^9^-THC formation in risk assessment of products for which there is not typically a hazard of decarboxylation (e.g., when the foods are intended for baking). It should be also considered that the more recent EFSA ARfD value is also based on ∆ ^9^-THC and not on total THC.
2. The reviewer is correct that risk assessment methods of mixtures are currently evolving and have not been applied to mixtures of cannabinoids. Typically, to provide such a risk assessment would be the responsibility of the food business operator and not the responsibility of the authority. Hopefully, such a risk assessment will be provided during the toxicological assessments necessary during the novel food application procedure, which several companies have initiated. In light of the currently available evidence, we do not believe a change in our risk assessment based on EFSA ARfD would be justified. For example, Solowij et al. ^2^ state that the effects of THC may even be enhanced by low-dose CBD (most food supplements would fall in the low CBD dose range) and may be particular prominent in infrequent cannabis users. Furthermore, Haney et al. ^3^ found that oral CBD does not reduce the reinforcing, physiological, or positive subjective effects of smoked cannabis. Niemsink and van Laar ^4^ acknowledge that CBD may counteract the negative effects of THC, but warned that the question remains how laboratory results translate to the real world. Furthermore, a recent survey reported a high prevalence of side effects in 74 out of 135 young adult cannabidiol users (55%), with dry mouth, feeling high, change in appetite and fatigue most commonly reported. ^5^ All in all, we strongly believe that the current evidence does not allow to negate side effects of THC in commercial cannabidiol products despite the potential antagonistic effect of CBD.
3. We agree with the reviewer that the German guidance value is typically one to several orders of magnitude lower than the THC contents in the products (please note that the guideline value is for THC. There is currently no guideline value for CBD available).

References:

1. Lachenmeier DW, Bock V, Deych A, *et al.*: Hemp food products - an update. *Deut Lebensm Rundsch.* 2019; **115**: 351–372. Publisher Full Text

2. Solowij N, Broyd S, Greenwood LM, van Hell H, et al.: A randomised controlled trial of vaporised Δ9-tetrahydrocannabinol and cannabidiol alone and in combination in frequent and infrequent cannabis users: acute intoxication effects. *Eur Arch Psychiatry Clin Neurosci*. 2019; **269** (1): 17-35 PubMed Abstract | Publisher Full Text 

3. Haney, M., Malcolm, R., Babalonis, S. *et al.* Oral Cannabidiol does not Alter the Subjective, Reinforcing or Cardiovascular Effects of Smoked Cannabis. *Neuropsychopharmacol* **41, **1974–1982 (2016). https://doi.org/10.1038/npp.2015.367

4. Niesink RJM and van Laar MW (2013) Does cannabidiol protect against adverse psychological effects of THC? Front. Psychiatry 4:130. https://doi.org/10.3389/fpsyt.2013.00130

5. Meghann Wheeler, Julie Williams Merten, Benjamin T Gordon & Hanadi Hamadi (2020) CBD (Cannabidiol) Product Attitudes, Knowledge, and Use Among Young Adults, Substance Use & Misuse, 55:7, 1138-1145, https://doi.org/10.1080/10826084.2020.1729201

# Reviewer report: Reviewer response for version 2

**DOI:** 10.5256/f1000research.24583.r59880

**Published:** 2020-02-17

**Authors:** Arno Hazekamp

**Affiliations:** 1 Hazekamp Herbal Consulting BV, Leiden, The Netherlands

The authors of this manuscript have addressed my questions and concerns in a satisfactory manner. I see no more objection for indexing of this very welcome manuscript.

Reviewer Expertise:

medicinal cannabis cultivation, quality control, development of administration forms, clinical trials, patient surveys.

I confirm that I have read this submission and believe that I have an appropriate level of expertise to confirm that it is of an acceptable scientific standard.

# Reviewer report: Reviewer response for version 1

**DOI:** 10.5256/f1000research.21875.r53494

**Published:** 2019-09-17

**Authors:** Linda A. Parker

**Affiliations:** 1 Department of Psychology, Neuroscience Graduate Program, University of Guelph, Guelph, ON, Canada

Commercial CBD products are usually crude extracts from whole hemp plant material, that are available for purchase in several venues. These extracts have been reported to contain cannabinoid mixtures rather than pure CBD, and are then mixed into edible oils to obtain CBD oil. They are marketed as being free of psychoactive component, i.e. THC. Anecdotal reports of THC-like side effects from these mixtures have been reported. Three hypotheses for these side effects are posed: i) direct pharmacological effect of CBD-for which there is little evidence, ii) the degradation of CBD to THC due to acidic hydrolysis in the stomach following oral consumption, and iii) THC directly contained in the products as a by-product due to co-extraction and enrichment or contamination. The article investigated the latter two of these hypotheses.

 CBD degradation: Differently concentrated CBD in methanolic solutions was evaluated in a range corresponding to typical amounts consumed in supplements based on commercial CBD supplied by Merck. These solutions were exposed to an artificial gastric juice at different incubation times and under different environmental conditions. In no case was there any conversion of CBD to THC in any of the samples. Indeed, if CBD is converted to THC in the stomach, among consumers taking CBD it would be expected that THC metabolites would be detectable in the blood and urine, but this has not been shown in oral CBD studies.

 THC contamination as a cause of side effects: A sampling of all available CBD products registered as food supplement in the German State Baden-Württemberg, other hemp extract products in retail, as well as products available at the warehouse of a large internet retailer were evaluated for THC content between December 2018 and July 2019. Of the 28 samples described in Table 2, none of the products was compliant with European food regulations and most of the samples contained THC, some at a dose that would be expected to lead to intoxication. Therefore, the results provided evidence that THC contamination in the CBD products is the most likely cause for the anecdotal THC-like side effects reported. Although it would have been even more informative to have a clear indication of the CBD content of each of the samples, the data clearly present evidence that the products are mislabeled and that THC-like side effects reported by patients is likely the result of contamination of the product with THC, which was the purpose of the study.

 This is an important manuscript that will clear up the misconception that CBD is converted to THC in gastric juices of users.

Is the work clearly and accurately presented and does it cite the current literature?

Yes

If applicable, is the statistical analysis and its interpretation appropriate?

Yes

Are all the source data underlying the results available to ensure full reproducibility?

Yes

Is the study design appropriate and is the work technically sound?

Yes

Are the conclusions drawn adequately supported by the results?

Yes

Are sufficient details of methods and analysis provided to allow replication by others?

Yes

Reviewer Expertise:

Cannabinoids, nausea, CBD, rat models, addiction, learning

I confirm that I have read this submission and believe that I have an appropriate level of expertise to confirm that it is of an acceptable scientific standard.

# Response

**Published:** 2020-01-31

**Authors:** Dirk W. Lachenmeier

**Affiliations:** Chemisches und Veterinäruntersuchungsamt (CVUA) Karlsruhe, Germany

Thank you for your assessment of our article.

# Reviewer report: Reviewer response for version 1

**DOI:** 10.5256/f1000research.21875.r52382

**Published:** 2019-08-19

**Authors:** Arno Hazekamp

**Affiliations:** 1 Hazekamp Herbal Consulting BV, Leiden, The Netherlands

The manuscript focuses on the quality of CBD oils, which is a meaningful and contemporary issue. Table 2 is the core of the study, because it compares the **claimed **composition of CBD oil, with **lab results** obtained by the authors. The conclusion is that the currently available products in Germany are often not what they claim to be.

 Unfortunately, the authors did not analyze the actual CBD content of many of the products, and they assume that their own lab analyses are fully accurate, without proving or showing why. The authors use two different methods of analysis without explaining why one method is not sufficient. Also, in many parts of the text, they explain the current situation concerning CBD product without realizing that many readers may not have enough background information to follow their line of reasoning. The manuscript should be rewritten to explain basic concepts better.

 Also, more data should be added to table 2, particularly about CBD content of the products analyzed. Right now, CBD analysis data is missing for more than half of the samples. It is not clear why so many of the products have not been studied for CBD content, and this undermines the strength of the paper. In general, the idea behind the study is very good, but the execution is relatively poor because it only focuses on the THC content of the product analyzed.

 Please see my annotated copy of the article here for additional comments.

Is the work clearly and accurately presented and does it cite the current literature?

Yes

If applicable, is the statistical analysis and its interpretation appropriate?

Partly

Are all the source data underlying the results available to ensure full reproducibility?

Partly

Is the study design appropriate and is the work technically sound?

Partly

Are the conclusions drawn adequately supported by the results?

Partly

Are sufficient details of methods and analysis provided to allow replication by others?

Partly

Reviewer Expertise:

medicinal cannabis cultivation, quality control, development of administration forms, clinical trials, patient surveys.

I confirm that I have read this submission and believe that I have an appropriate level of expertise to confirm that it is of an acceptable scientific standard, however I have significant reservations, as outlined above.

# Response

**Published:** 2020-01-31

**Authors:** Dirk W. Lachenmeier

**Affiliations:** Chemisches und Veterinäruntersuchungsamt (CVUA) Karlsruhe, Germany

Thank you for your detailed comments and annotations in the copy. As requested, we have revised the background information to clarify the basic concepts.

Regarding the criticism of lack of CBD analysis, it must be remarked that the aim of our paper was to investigate the side effects of the products due to THC contamination. Hence, the main purpose of our analytical efforts was to accurately determine the content of THC for health risk assessment. See also the title of the paper, which is regarding THC and not CBD. The analysis of CBD is more or less a secondary addition to the aim of our study, which was THC analysis. It is therefore true that CBD quantification is missing for many samples for the pure reason that CBD and THC contents are so different and CBD was outside the linearity of our calibration. For cost reasons, we have refrained from determining CBD using a second method or dilution (it is of note that we had not specific funding for this study and have to generally work economically as tax-payer funded institute). In the legal evaluation of the products, the CBD content is more or less unimportant as long as the content is below the level of pharmacological action (for food products). As all products had to be objected for various reasons (lack of novel food authorisation, THC contents outside of acceptable levels, mandatory labelling etc.), the CBD quantification was not relevant as well because the issue of consumer deception by mislabelling of CBD is secondary to the safety aspects posed by THC or the use of non-approved, potentially unsafe novel food ingredients.

Regarding the question on analytical methods, we actually have confidence in our analytical methods and they are fully validated and our institute is externally accredited according to ISO 17025. Nevertheless, as there is no official method for CBD analysis available, we have confirmed our results with a second procedure to even further improve confidence and validity. As of now, we believe that both methods perform similarly and could both be used in instances of laboratories without access to two different instruments.

To improve the strength of the paper, as requested by the reviewer, we have added the results of 39 samples measured in the meantime (new total 67 samples). In many of these samples it was also possible to quantify CBD. The measurement of these additional samples corroborates our previous results and interpretation, and we hope that the sample collective now appears as sufficient for publication.

Regarding the comments in the annotated copy, we have revised the text considering all suggested changes, except for the following comments for which we provide a detailed response (comment numbering according to Adobe Acrobat comment numbering in annotated copy of reviewer):

- Page 3, comment #2 “Not yet. The European Food Safety Authority (EFSA) has advised that CBD should be classified as a novel food. But now it is up to individual EU member states to implement that advise into national legislation. Some countries may decide to not follow the advise.”

We disagree with this comment. The classification of CBD and hemp extracts (which was published in the novel food catalogue of the European commission and not by EFSA, see: https://ec.europa.eu/food/safety/novel_food/catalogue/search/public/index.cfm?ascii=Cannabinoids) is a consensus decision of all EU member states. EU regulations such as the novel food regulation are binding in its entirety and directly applicable in all Member States. Therefore there appears to be no leverage for member states to act in infringement of the novel food regulation. If you check the  Rapid Alert System for Food and Feed (RASFF) portal for CBD ( https://webgate.ec.europa.eu/rasff-window/portal/?event=SearchByKeyword&NewSearch=1&Keywords=cbd), there are more than 80 notifications of CBD products as „unauthorised novel food ingredient“ from various countries including Spain, Belgium, Denmark, Germany, Austria,  Switzerland, Slovenia, Lithuania, Italy, Sweden. In Germany, there are currently at least 7 court rulings that confirmed the status of CBD as novel food and confirmed the actions of the authorities (typically removal of products from the market).

For details on novel food status and German court rulings, please refer to: Lachenmeier DW, Rajcic de Rezende T, Habel S, et al.: Recent jurisdiction confirms novel food status of hemp extracts and cannabidiol in foods – Classification of cannabis foods under narcotic law is still ambiguous. Deut Lebensm Rundsch. 2020;116: 111-119. DOI: https://doi.org/10.5281/zenodo.3631608

The following court rulings confirmed the novel food status of cannabidiol and hemp extracts:

VG Cottbus 08.01.2020 Az. 3 L 230/19

OVG Lüneburg 12.12.2019 Az. 13 ME 320/19

VG Hannover 18.11.2019 Az. 15 B 3035/19

VG Gießen 11.11.2019 Az. 4 L 3254/19.GI

VGH Baden-Württemberg 16.10.2019 Az. 9 S 535/19

VG Düsseldorf 27.09.2019 Az. 16 L 2333/19

VG Stade 05.09.2019 Az. 6 B 735/19

 

- Page 5, comment #5: “Based on your table, this product seems to be the most reliable. But in fact this sample may not contain any cannabinoids at all.”

Some cannabinoids could be qualitatively detected in this sample around the detection limit of the method.

- Page 4, comment #1: “It is not common to use two methods and use the average. Does that mean you do not trust your own methods?”

In our line of work in providing expert opinions that may be used in court cases, it is often common to use two methods, especially in cases where a reference procedure is not established or when there may be grave consequences in application of the results, such as taking products from the market. We currently cannot see the reason why doing more than perhaps absolutely necessary might hinder publication of such results.

Furthermore, as there was a discrepancy between our results and some previous studies regarding *in vitro* formation of THC from CBD, we found it prudent to confirm our results using a second methodology.

- Page 4, comments #3 and #8 regarding THCA, CBDA and CBN

Basically, we can accurately quantify all these other cannabinoids using the same method. However, as the results of these are not presented and unnecessary for the current paper, we have deleted all mentions of these compounds in the method section to avoid confusion.

- Page 5, comment #7: “Why are some samples measured 1 time, and others up to 6 times?”

The number of replicates depended on several factors, sometimes restricted by the very low sample volume we have received. Typically in the cases with highest THC content leading to a judgment of “non-safe food product” we aimed for at least 3 if possible 5 replicates. In certain cases, more replicates were made, for example when several dilutions were within the linearity range.
